# Characterization of the human RFX transcription factor family by regulatory and target gene analysis

**DOI:** 10.1186/s12864-018-4564-6

**Published:** 2018-03-06

**Authors:** Debora Sugiaman-Trapman, Morana Vitezic, Eeva-Mari Jouhilahti, Anthony Mathelier, Gilbert Lauter, Sougat Misra, Carsten O. Daub, Juha Kere, Peter Swoboda

**Affiliations:** 1 Department of Biosciences and Nutrition, Karolinska Institutet, Huddinge, Sweden; 2 Department of Biology, Bioinformatics Centre, Section for Computational and RNA Biology, University of Copenhagen, Copenhagen, Denmark; 3 Department of Medical Genetics, Centre for Molecular Medicine and Therapeutics at the Child and Family Research Institute, University of British Columbia, Vancouver, Canada; 4 Centre for Molecular Medicine Norway (NCMM), Nordic EMBL partnership, University of Oslo, Oslo, Norway; 5 Department of Cancer Genetics, Institute for Cancer Research, Oslo University Hospital Radiumhospitalet, Oslo, Norway; 6 Department of Laboratory Medicine, Karolinska Institutet, Huddinge, Sweden; 7 Science for Life Laboratory, Karolinska Institutet, Stockholm, Sweden; 8 School of Basic and Medical Biosciences, King’s College London, London, UK; 9 Folkhälsan Institute of Genetics and Molecular Neurology Research Program, University of Helsinki, Helsinki, Finland

**Keywords:** Cell differentiation, Cilia, Spermatogenesis, Immune cell proliferation, Neuronal development, Cell cycle control, Tumor suppression

## Abstract

**Background:**

Evolutionarily conserved RFX transcription factors (TFs) regulate their target genes through a DNA sequence motif called the X-box. Thereby they regulate cellular specialization and terminal differentiation. Here, we provide a comprehensive analysis of all the eight human *RFX* genes (*RFX1–8*), their spatial and temporal expression profiles, potential upstream regulators and target genes.

**Results:**

We extracted all known human *RFX1–8* gene expression profiles from the FANTOM5 database derived from transcription start site (TSS) activity as captured by Cap Analysis of Gene Expression (CAGE) technology. *RFX* genes are broadly (*RFX1–3, RFX5, RFX7*) and specifically (*RFX4, RFX6*) expressed in different cell types, with high expression in four organ systems: immune system, gastrointestinal tract, reproductive system and nervous system. Tissue type specific expression profiles link defined RFX family members with the target gene batteries they regulate. We experimentally confirmed novel TSS locations and characterized the previously undescribed *RFX8* to be lowly expressed. *RFX* tissue and cell type specificity arises mainly from differences in TSS architecture. *RFX* transcript isoforms lacking a DNA binding domain (DBD) open up new possibilities for combinatorial target gene regulation. Our results favor a new grouping of the RFX family based on protein domain composition. We uncovered and experimentally confirmed the TFs SP2 and ESR1 as upstream regulators of specific *RFX* genes. Using TF binding profiles from the JASPAR database, we determined relevant patterns of X-box motif positioning with respect to gene TSS locations of human RFX target genes.

**Conclusions:**

The wealth of data we provide will serve as the basis for precisely determining the roles RFX TFs play in human development and disease.

**Electronic supplementary material:**

The online version of this article (10.1186/s12864-018-4564-6) contains supplementary material, which is available to authorized users.

## Background

RFX (Regulatory Factor binding to the X-box) transcription factors (TFs) share and are defined by a conserved, specialized winged-helix type DNA binding domain (DBD) [1]. *RFX* genes have been identified in all animals within the Unikont branch of eukaryotes, which excludes algae, plants and various protozoan branches [2]. Metazoan genomes encode one to several *RFX* genes. *C. elegans* possesses one, *Drosophila* has two [3, 4], mammals have eight and – due to genome duplication – fishes have nine *RFX* genes [2, 5–10]. Human *RFX1–7* have previously been described [9], while *RFX8* (ENSG00000196460, www.ensembl.org) has not been characterized.

In different organisms, RFX TFs have been shown to regulate genes involved in various and seemingly disparate cellular and developmental processes [7] like the cell cycle and DNA repair [11, 12], or aspects of cellular differentiation, like the functional maturation of cells of the immune response [13] and the development of cilia on the surface of polarized cells [14–16]. As a consequence of these roles in development, mutations in *RFX* genes can lead to severe disease states. Mutations in *RFX5* cause autosomal recessive Bare Lymphocyte Syndrome (OMIM #209920), characterized by severe combined immunodeficiency due to failure in HLA expression. Mutations in *RFX6* cause autosomal recessive Mitchell-Riley Syndrome, characterized by neonatal diabetes and malformations of the gut (OMIM #615710). *Rfx* mutant mice exhibit a plethora of mild to fatal phenotypes, ranging from male sterility [17] to brain abnormalities [18]. These phenotypes are often attributed to cilia dysfunction [17, 19–21]. Of note, many human ciliopathy genes are strongly assumed to be RFX TF targets, given that their orthologs have been shown to be RFX TF targets in several different organisms, ranging from *C. elegans* to mouse [22–24].

In addition to the DBD, RFX TFs may contain other conserved domains like activation (AD) and dimerization (DIM) domains and the domains B and C of unknown function [7, 9]. The RFX DBD recognizes an imperfect inverted repeat sequence, the X-box motif, to which it binds [1]. RFX TF binding to the X-box motif has repeatedly been demonstrated by using methods ranging from in vitro binding studies, in vivo expression and mutation analyses to SELEX and ChIP sequencing approaches [8, 25–28]. Combined, these approaches led to the discovery of large batteries of RFX target genes [16].

By contrast, very little is known about upstream regulators of *RFX* genes. So far only a few studies in mice, zebrafish and flies have identified TFs of the bHLH class, Neurog3 and Atonal, as well as the homeobox protein Noto as upstream regulators of *RFX* genes [29–31]. In the yeast *S. cerevisiae* an upstream phosphorylation cascade controls expression of the RFX gene Crt1 [32].

In this study – using extensive analysis of data from the FANTOM5 database followed by experimental validations – we present an in-depth characterization of the entire human *RFX* gene family (*RFX1–8*), including the previously undescribed *RFX8* and *RFX* transcript isoforms that encode TFs without DBD. We provide an updated grouping of human RFX TFs and show that RFX functional domain composition is independent of expression profile. Our exhaustive analysis of *RFX* expression in many different human tissues and cell types suggests that *RFX* tissue and cell type specificity arises mainly from differences in TSS architecture and not from different transcript isoforms. We determined with high precision the positioning of X-box motifs with respect to TSS locations of human RFX target genes. Using cluster analysis based on tissue and cell type specific expression profiles we link defined RFX family members with the target gene batteries they regulate. Further, we provide a first list of candidate upstream regulators of human *RFX* genes. The wealth of data we provide will serve as the basis for future studies of the role of RFX TFs in human development and disease.

## Results

### Expression of human *RFX* genes in different tissue types

Detailed expression profiles of the human *RFX1–8* genes have not been described. We used data from the FANTOM5 database that is based on experimental expression profiling by CAGE technology across a wide spectrum of human biological samples. The expression level of a given CAGE TSS location is defined by an arbitrary unit, tags per million (TPM) [33]. We extracted 37 CAGE TSS locations for *RFX1–8* from the FANTOM 5database and shortlisted these to 30 TSS locations by merging those which are in close proximity to each other and have similar expression profiles (cf. Methods). We then named these 30 TSS locations alphabetically, whereby promoter A (pA) is the highest expressed TSS. Expression of each *RFX* TSS is described in detail for human tissues, primary cells and cell lines (Additional file 1). The wealth of biological samples allows classifying the expression profiles for human *RFX1–8* in different cell types.

A given *RFX* TSS location is considered as being expressed broadly if it is expressed at TPM > 5 in a large number and variety of tissues (*n* > 10). Conversely, a given *RFX* TSS location is considered as being expressed specifically if it is expressed at TPM > 5 in a small number of tissues of the same organ (*n* < 10). We found most TSS locations of *RFX1–3, 5* and *7* to be expressed broadly in many tissue types whereas the TSS locations of *RFX4* and *RFX6* are all expressed in specific tissue types. pA@RFX4 is highly specific in brain and spinal cord tissues, while pB and pC@RFX4 are highly specific in testis. *RFX6* TSS locations are all specifically expressed in the gastrointestinal tract (GI) (Additional file 2). We performed hierarchical clustering of the 30 RFX TSS locations based on their expression values (TPM) across 135 human tissue samples. Thereby we identified four major tissue clusters, namely immune system [34], gastrointestinal tract [35, 36], testis [37] and brain and spinal cord [18, 38–41], and two minor clusters, namely uterus and lung [42] (Additional file 2).

The expression of *RFX8* is very low, making it the most elusive member of the human RFX family that has hitherto avoided detection. Here we identified *RFX8* TSS locations with highest expressions in some tissues of the immune system (pA, pC, pD) and the gastrointestinal tract (pB, pE). However, the tissue expression values for pC, pD and pE were hard to distinguish from background noise (TPM < 1). In primary cells and cell lines, *RFX8* TSS locations had higher expression values, with the most prominent expression in a Schwannoma cell line (Additional file 2).

### Connecting TSS expression profiles to protein-coding transcript isoforms

FANTOM5 data allowed us to determine TSS locations and expression profiles. In order to connect the 30 *RFX* TSS locations described above to known transcript isoforms, we set a maximum distance limit of 50 nt between the TSS location and the nearest Ensembl protein-coding transcript with a complete open reading frame. We found that 18 of these 30 *RFX* TSS locations matched Ensembl protein-coding transcripts. The remainder (12) of the 30 *RFX* TSS locations were treated as novel transcript isoforms in human tissues. We selected seven of these as representatives for experimental validation by RT-PCR and sequencing (Table 1, Additional file 3: Table S1). The seven novel transcripts consist of **(i)** testis specific pC@RFX1 and pE@RFX3, **(ii)** broadly expressed and highest in brain pC@RFX3, pC@RFX5, pA@RFX7, pC@RFX7, and **(iii)** lowly expressed pA@RFX8.

**Table 1:** *RFX1–8* expression data and novel transcripts

Gene (chromosome)	TSS	Matched Ensemble transcript	Tissue profile summary	
			Expression	Highest in
*RFX1* (chr19)	pA@RFX1	ENST00000254325	Broad	Cerebellum (brain)
	pB@RFX1	ENST00000254325		
	pC@RFX1*	Novel transcript*	Specific	Testis
*RFX2* (chr19)	pA@RFX2	ENST00000303657	Broad	Uterus
	pB@RFX2	ENST00000303657		Testis
	pC@RFX2	ENST00000303657		Medulla oblongata (brain)
*RFX3* (chr9)	pA@RFX3	ENST00000382004	Broad	Cerebellum (brain)
	pB@RFX3	ENST00000382004		Lung, fetal
	pC@RFX3*	Novel transcript*		Cerebellum (brain)
	pD@RFX3	ENST00000382004		Lung, fetal
	pE@RFX3*	Novel transcript*	Specific	Testis
*RFX4* (chr12)	pA@RFX4	ENST00000392842	Specific	Spinal cord
	pB@RFX4	ENST00000229387	Specific	Testis
	pC@RFX4	ENST00000357881		
*RFX5* (chr1)	pA@RFX5	ENST00000290524	Broad	Blood (immune system)
	pB@RFX5	ENST00000290524		Tonsil (immune system)
	pC@RFX5*	Novel transcript*		Brain, fetal
	**pD@RFX5**	**Novel transcript**		Duodenum, fetal (GI)
*RFX6* (chr6)	pA@RFX6	ENST00000332958	Specific	Duodenum, fetal (GI)
	pB@RFX6	ENST00000332958		
	pC@RFX6	ENST00000332958		
*RFX7* (chr15)	pA@RFX7*	Novel transcript*	Broad	Cerebellum (brain)
	pB@RFX7	ENST00000559447		
	pC@RFX7*	Novel transcript*		
	**pD@RFX7**	**Novel transcript**		
*RFX8* (chr2)	pA@RFX8*	Novel transcript*	Lowly expressed (TPM < 5)	Thymus (immune system)
	pB@RFX8	ENST00000428343		Medial frontal gyrus (brain)
	**pC@RFX8**	**Novel transcript**	Noise (TPM < 1)	Heart
	**pD@RFX8**	**Novel transcript**		Breast
	**pE@RFX8**	**Novel transcript**		Rectum, fetal

Thirty TSS locations from eight human *RFX* genes and their respective tissue profile summaries are presented (cf. Methods; GI = gastrointestinal tract). For an expanded summary and the analysis of functional domains, see Tables S1 and S2 in Additional file 3, respectively. Novel transcripts are marked in bold and those selected for experimental validation are marked with an asterisk. For RT-PCR verified sequences of novel transcripts, see Table S9 in Additional file 3

**Fig. 1:**
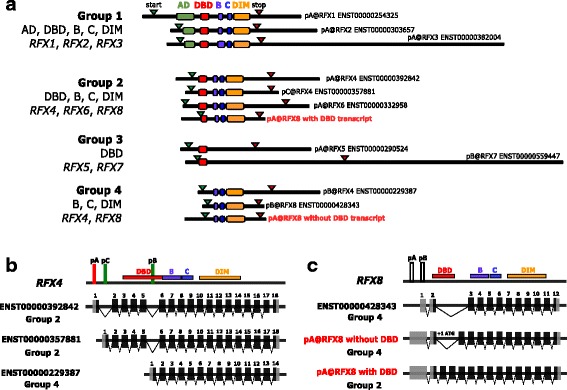
Representative *RFX* transcripts grouped according to their functional domain compositions. **a** Representative *RFX* transcripts (to scale in nucleotides / nt) can be categorized based on the presence or absence of functional domains. Group 1 consists of *RFX1, RFX2,* and *RFX3*, which have all the domains. Group 2 consists of *RFX4, RFX6* and *RFX8*, which have all domains but the AD. Group 3 consists of *RFX5* and *RFX7*, which have only the DBD. Group 4 is novel, consisting of isoforms of *RFX4* and *RFX8*, which lack the DBD. The start of the black bar marks the TSS position. Green and red arrows mark start and stop codon positions, respectively. The RFX protein domains encoded by these transcripts are AD (activation domain), DBD (DNA binding domain), B (domain B), C (domain C), and DIM (dimerization domain). They are indicated using color-coded boxes. The DBD (red box), which typically spans 222–225 nt (cf. Table S2 in Additional file 3) serves as a size marker. **b**, **c** *RFX4* and *RFX8* TSS locations illustrate best that RFX functional domain composition is independent of expression profile. They are connected to Ensembl protein-coding transcripts or shown as novel, validated transcripts (in red). Exon numbers refer to those in the corresponding Ensembl transcript IDs (distance and positions are not to scale). pA@RFX4 (red) belongs to the brain and spinal cord cluster, whereas pB and pC@RFX4 (green) belong to the testis cluster (cf. Additional file 2). The highest expressed tissues for pA and pB@RFX8 are thymus and medial frontal gyrus, respectively, and they are not color-coded because of their low expression levels in tags per million (TPM < 5) (Table 1)

Next, we assessed the full transcript sequences (from 5′ to 3’ UTRs) including their coding potential (from start to stop codons) from both the matched Ensembl protein-coding transcripts and the novel sequence-verified transcripts (Table S2 in Additional file 2). Representatives of the *RFX1–8* transcripts are shown in Fig. 1a. We found that the majority of RFX transcript isoforms originating from the same gene encode identical proteins suggesting that tissue and cell type specificity arises mainly from differences in TSS architecture and regulation through their corresponding promoters. The exceptions are *RFX1*, *RFX4* and *RFX8* with isoforms encoding different protein variants. The testis specific pC@RFX1 transcript isoform encodes a shortened N-terminal region upstream of the activating domain (AD). RFX4 transcript isoforms have been extensively studied [43–45] and thus complement our results, where we found isoforms encoding different RFX4 protein variants. The *RFX8* gene encodes a TF protein lacking a DNA binding domain (DBD) (ENSP00000401536, www.ensembl.org). Here, we experimentally validated *RFX8* transcripts by sequencing cDNA from human brain total RNA and uncovered novel splicing patterns leading to alternative RFX8 protein variants, with and without DBD (Table S1 in Additional file 3).

### RFX functional domain composition is independent of expression profile

Human RFX TFs were previously categorized through phylogenetic analysis of their four functional domains outside the DBD: activating domain (AD), domain B, domain C and dimerization domain (DIM) [9, 10, 16]. Given the variation in coding potential of all 30 *RFX1–8* transcript isoforms, we investigated whether there is a correlation between the presence or absence of certain RFX functional domains and the CAGE TSS expression profiles in human tissues. First, we categorized all *RFX1–8* transcripts into four groups based on their functional domain structure: **(i)** Group 1: *RFX1–3* with all known domains, **(ii)** Group 2: *RFX4, RFX6* and *RFX8* lacking the AD, **(iii)** Group 3: *RFX5* and *RFX7* with only the DBD, **(iv)** Group 4: *RFX4* and *RFX8* lacking the DBD (Fig. 1a). When we then compared these four groups to their respective TSS expression profiles, we did not find any indication that RFX TFs with similar domain composition would be expressed broadly or specifically in a certain tissue cluster, suggesting that the RFX functional domain composition is independent of expression profile. We analyzed *RFX4* and *RFX8* in more detail to illustrate this point (Fig. 1b, c).

Based on the FANTOM5 expression profiles, the gene *RFX4* is highly tissue specific compared to other *RFX* genes. In our analysis, we connected three *RFX4* TSS locations to three different Ensembl protein-coding transcripts (Fig. 1b). The longest, highest expressed transcript falls into Group 2 (lacking the AD); it is specifically expressed in the brain and spinal cord. The other two less expressed transcripts are both testis specific, whereby one belongs to Group 2 (lacking the AD) and the other one to Group 4 (lacking the DBD). The newly described gene *RFX8* is the least expressed of all the *RFX* genes. We connected the two highest expressed *RFX8* TSS locations with three possible transcripts (Fig. 1c). The same TSS can lead to different transcripts encoding protein variants, which either fall into Group 2 (lacking the AD) or Group 4 (lacking the DBD), suggesting an additional layer of gene regulation on top of the TSS architecture itself. Interestingly, *RFX8* transcripts with DBD revealed that the RFX8 DBD is slightly shorter than the DBDs in RFX1–7. Whereby, multiple sequence alignments of RFX DBD amino acid sequences reveal that it is the least conserved N-terminal amino acids of the DBD that are missing in RFX8 (Figure S1 in Additional file 3).

### Tissue and cell type specific clustering of RFX family members with the target genes they regulate

**Fig. 2:**
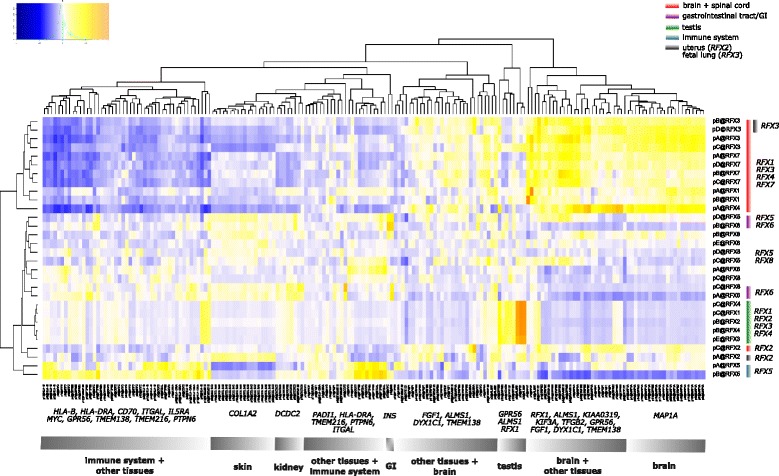
Heat map of tissue expression clusters of *RFX1–8* and their experimentally confirmed target genes in humans. Heat map of unsupervised hierarchical clustering of 30 TSS locations of *RFX* genes and 185 TSS locations of validated RFX target genes (with shorthand p for promoter) based on the expression values in tags per million (TPM) across 135 human tissue samples extracted from FANTOM5. The heat map color-code represent Pearson correlation values with a gradient of − 1 in dark blue/blue (negative correlation), 0 in white (zero correlation) and 1 in yellow/orange (positive correlation). The graph was generated by the heatmap.2::gplots [95] *R* package. *RFX* TSS locations tissue clusters (y-axis) are color-coded as described in Additional file 2. The tissue cluster divisions of RFX target genes (x-axis) are based on groups of tissues with the highest expression values (TPM) of the respective TSS locations. The term “other tissues” includes adipose, kidney, lung, seminal vesicle, skeletal muscle, throat and uterus

To correlate and eventually predict which RFX family member regulates which target gene in which human tissue and cell type, we compared and clustered the expression profiles of all RFX family members with direct RFX target genes. We selected from the literature a large number of validated direct RFX target genes in humans, as demonstrated either by a biochemical interaction between an RFX TF and the respective X-box promoter motif or by confirmation of the X-box function by mutation analysis (Table S3 in Additional file 3). We then extracted the CAGE TSS expression values (TPM) of these genes from the FANTOM5 database and performed unsupervised heat map clustering based on the correlations of expression values across 135 tissue types (Fig. 2).

We identified strong tissue specific *RFX* and target gene clusters, namely for testis (*RFX1–4* and target genes *GPR56*, *ALMS1*, *RFX1*) and the gastrointestinal tract (*RFX5–6* and target gene *INS*/insulin). We also observed strong differences between clusters of the immune system (*RFX5*) and the brain (*RFX1, 3, 4, 7*). This underscores that **(i)** the respective RFX family members regulate different sets of target genes as they are not co-expressed in a given tissue type, and **(ii)** for a given target gene RFX TFs can act as activators or as inhibitors. For brain tissue and cell types, *RFX1, 3, 4* and *7* clustered tightly together, indicating a preference for these RFX family members to (co-) regulate the expression of brain-specific genes such as the ciliopathy/Alström syndrome gene *ALMS1*, the dyslexia candidate gene *KIAA0319* or the gene *MAP1A*. Interestingly, another member of the brain cluster, pC@RFX2 (Table 1, Additional file 2), in the context of target genes clustered separately (Fig. 2), suggesting that in the brain RFX2 regulates a distinct set of target genes. Alternatively, RFX2 may interact with other RFX family members or other co-factors without preference as long as they are co-expressed in a given tissue and cell type.

### X-box motif positioning in the human genome

RFX TFs regulate their target genes by binding to a conserved X-box motif in the promoter region. Previous X-box motif searches have typically been carried out using 1–3 kb sequence windows upstream of the TSS or ATG, such as in *C. elegans* [27], *D. melanogaster* [23], mouse and human [46]. To our knowledge, precise X-box motif positioning has not been characterized in the human genome. Thus, we determined the most likely positioning of functional X-box motifs in the promoter region, defined as 5000 bp upstream (− 5000) and 2000 bp downstream (+ 2000) in relation to TSS locations, of experimentally validated human RFX target genes.

**Fig. 3:**
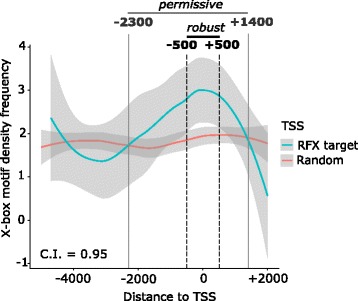
X-box motif position with respect to TSS locations. Density frequency of X-box motif positions with respect to the TSS locations of experimentally proven direct RFX target genes in humans (shown in blue) and a set of 10× random TSS locations from FANTOM5 (shown in red): TSS -5000 to + 2000 bp windows were scanned with two JASPAR RFX motifs (RFX2 MA0600.1 and RFX5 MA0510.1) with 80% threshold. We define a sequence window as “robust” by the area where the two curves with 95% C.I. smoothing do not overlap. We define a sequence window as “permissive” by the area where the two curves intersect

To facilitate the search, we used two curated TF binding profiles for human RFX available in the JASPAR (2018) database [47]: RFX2 (MA0600.1) and RFX5 (MA0510.1) (Table S4 in Additional file 3). As a control, we selected a 10-fold larger random set of TSS locations across the human genome. Our search effort revealed that X-box hits are typically located very close to RFX target genes TSS locations (Fig. 3, Table S5 in Additional file 3). Based on search and find statistics the X-box positioning window can be further subdivided into a robust window of − 500 to + 500 bp and a permissive window of − 2300 to + 1400 bp. Using an independent search approach (the MEME suite FIMO software) [48] we confirmed these overall search and find parameters for human X-box motifs. Our analysis enhances the prediction power of future searches for functional X-box motifs, which relates to both upstream *and* downstream of TSS locations of candidate human RFX target genes and pinpoints their likely locations. Functional X-box motifs at larger distances from TSS locations (e.g at distal enhancers) are likely to be the exception rather than the norm (Table S6 in Additional file 3).

### Prediction of upstream RFX regulators using transcription factor binding site (TFBS) analysis

Identifying the upstream regulators of *RFX* genes will allow predicting the developmental and cellular niche that RFX TFs occupy. Thus, we searched for TF binding profiles over-represented in the promoter and enhancer regions of all 8 human *RFX* genes. We used search windows of − 5000 to + 2000 bp in relation to 30 *RFX* TSS locations and - 200 to + 200 bp around the midpoints of 13 significantly correlated candidate *RFX* enhancer sequences (extracted from Andersson et al. [49]; Table S7 in Additional file 3). We then scanned these regions with all the core vertebrate TF binding profiles present in the JASPAR 2016 database [47]. The enrichment for TF binding profiles was assessed against a 10-fold larger random set of human promoter and enhancer regions using the oPOSSUM3 tool [50].

**Fig. 4:**
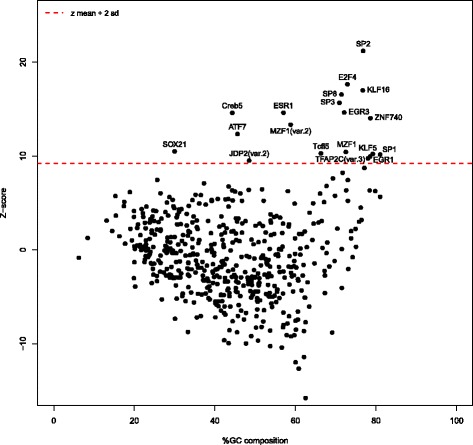
TF binding profiles in the promoter and enhancer regions of *RFX* genes. Distribution of all the z-scores of all the core vertebrate transcription factor binding site (TFBS) profiles in JASPAR 2016, with the search areas consisting of − 5000 to + 2000 bp with respect to the 30 *RFX* TSS locations and − 200 bp to + 200 bp from the mid-points of the *RFX* enhancers, against a background of a set of 10× random TSS locations and enhancers with identical window size and matching %GC distribution from FANTOM5. High-scoring or over-represented TF binding site profiles were computed as having z-scores above the mean + 2 x standard deviation (red dotted line)

We identified 19 over-represented TF binding profiles (Fig. 4) associated to the TFs SP2 (*specificity protein 2*) (JASPAR profile MA0516.1), E2F4 (*E2 factor 4*) (MA0470.1), KLF16 (*Kruppel like factor 16*) (MA0741.1), SP8 (*specificity protein 8*) (MA0747.1), SP3 (*specificity protein 3*) (MA0746.1), EGR3 (*early growth response 3*) (MA0732.1), ESR1 (*estrogen receptor alpha*) (MA0112.3), Creb5 (*cAMP responsive element binding protein 5*) (MA0840.1), ZNF740 (*zinc finger protein 740*) (MA0753.1), ATF7 (*activating transcription factor 7*) (MA0834.1), SOX21 (*sex determining region Y-box 21*) (MA0866.1), MZF1 *(myeloid zinc finger 1)* (MA0056.1 and MA0057.1), Tcfl5 (*transcription factor like 5*) (MA0632.1), KLF5 (*Kruppel like factor 5*) (MA0599.1), SP1 (*specificity protein 1*) (MA0079.3), EGR1 (*early growth response 1*) (MA0162.2), TFAP2C (*transcription factor AP-2 gamma*) (MA0815.1) and JDP2 (*Jun dimerization protein 2*) (MA0656.1). The full list of the TF binding profiles can be found in Additional file 4.

### siRNA validation of RFX regulators

**Fig. 5:**
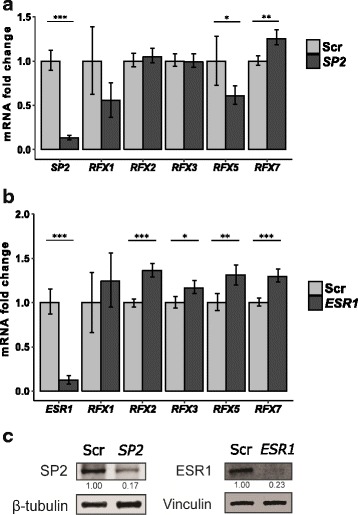
siRNA validation of candidate *RFX* regulators. The genes *SP2* and *ESR1* represent the high-scoring group of candidate *RFX* regulators (cf. Fig. 4 and Additional file 4). In the MCF7 breast cancer cell line, amplification efficiency-adjusted mRNA fold change quantifications of *RFX1, 2, 3, 5* and *7* were normalized to the geometric mean of *HPRT1* and *HSPCB*, whereby a fold change equaling 1 describes an unchanged expression level. For this we used (**a**) *SP2* siRNA versus scrambled (Scr) control siRNA knockdown, and (**b**) *ESR1* siRNA versus scrambled (Scr) control siRNA knockdown. In (**a**, **b**) error bars represent SEM and fold-change statistical significance was calculated using the student two-sample t-test (***p-value ≤0.01, **p-value ≤0.05, *p-value ≤0.1). **a**, **c** *SP2* siRNA knockdown was confirmed at both the mRNA and protein level and a significant up-regulation of *RFX7* and down-regulation of *RFX5* were observed. **b**, **c** *ESR1* siRNA knockdown was confirmed at both the mRNA and protein level and significant up-regulations of *RFX2, 3, 5* and *7* were observed. **c** Immunoblotting band intensities were quantified using ImageJ and normalized with the indicated loading controls

To test if any of the over-represented TF binding profiles can be linked to functional upstream regulation of *RFX* genes, we selected two TFs within the high-scoring TF binding profiles. We used siRNA knockdown of *SP2* and *ESR1* followed by qRT-PCR measuring the fold change of mRNA expression levels of *RFX* genes. We could successfully demonstrate knockdown of *SP2* and *ESR1* both at the mRNA level by qRT-PCR and at the protein level by immunoblotting (Fig. 5). The TF binding profiles of SP2 and ESR1 both scored clearly above the z-score threshold (Fig. 4). We selected the human MCF7 breast cancer cell line for which data are available in FANTOM5. In this cell line the genes *SP2*, *ESR1, RFX1–3, − 5,* and *− 7* are expressed sufficiently high (TPM > 5), while the genes *RFX4*, *− 6* and − *8* are not expressed (TPM = 0). We used efficiency-adjusted fold change quantification against scrambled (Scr) control siRNA normalized to the geometric mean of *HPRT1* and *HSPCB* as two independent reference genes [51]. All the Ct levels of the test siRNA and Scr control siRNA can be found in Additional file 5.

We observed that siRNA knockdown of *SP2* and *ESR1* in human MCF7 cells resulted in a significant fold change in the mRNA expression levels of at least one of the *RFX* genes (Fig. 5). siRNA knockdown of *SP2* resulted in both activating and inhibiting effects on *RFX* genes, whereby only *RFX7* showed significant up-regulation. In contrast, siRNA knockdown of *ESR1* revealed consistent inhibitory effects on all the *RFX* genes analyzed, with *RFX2, − 3, − 5*, and *− 7* being significantly up-regulated. These data show that computational TFBS analyses of the promoter regions of *RFX1–8* correctly identified functional upstream regulators of these *RFX* genes. Depending on the individual *RFX* gene these upstream regulators act either as activators or as repressors.

## Discussion

We have exhaustively analyzed all eight members of the human RFX TF gene family (*RFX1–8*). By extracting and computationally analyzing large-scale experimental data sets, we were able to describe in detail *RFX* gene expression as well as the *RFX* gene regulatory landscape in many different human tissues and cell types, including in parts the experimental validation thereof. We provide the first detailed experimental characterization of *RFX8* and of *RFX* isoforms without DBD. Further, we provide insight into upstream regulators of human *RFX* genes and determined the sequence windows in which – in most cases – human RFX TFs act as direct regulators of their target genes. Thereby we provide an in-depth catalogue and key resource for future work on the roles that RFX TFs play in human development and disease.

Our extensive survey of all the human *RFX (1–8)* gene expression profiles enabled us to carefully analyze all the transcript isoforms and determine the potential protein variants encoded by these isoforms. We ordered all expression profiles from low to high, from broad to tissue specific, made tissue and cell type assignments, including isoform correlations and non-correlations, and thereby were able to cluster the expression profiles for all the isoforms of all the human *RFX* genes. We found and experimentally validated that – typically – *RFX* gene TSS locations (of the same gene) would lead to the same protein variant, suggesting that it is mostly promoter and TSS architecture that gives rise to diversity in gene expression profiles. These results highlight the importance of studying non-coding regulatory regions of key genes involved in developmental processes such as cell type specification and differentiation. Exceptions include TSS locations for the gene *RFX4* that were spread across a large genomic distance leading to transcript isoforms encoding different tissue specific protein variants.

Our work lead to an updated grouping of human RFX TFs and showed that RFX functional domain composition is independent of expression profile. We identified two *RFX* genes, *RFX4* and *RFX8*, which can encode protein variants without DBD. The function of RFX protein variants without DBD is unclear. Possibly, they act as tissue-specific co-repressors, similar to SHP proteins [52]. This potential role is clearly inferred for RFX4, where such competitive co-repression may occur in the testis but not in the nervous system [44]. Transcript validation for the newly described gene *RFX8* revealed the possibility for encoding protein variants with and without a DBD. For the protein variant with DBD, this domain would be slightly shorter as it is missing the least conserved N-terminal 20 amino acids. In addition to the overall low expression level of *RFX8*, it raises the question of RFX8 functionality. *RFX8* was most prominently expressed in Schwannoma cells, suggesting a role for RFX8 in Schwann cell proliferation.

Given the central role that RFX TFs play during development (e.g. in the differentiation of cilia), we were interested in finding candidate upstream regulators of *RFX* genes. We used computational predictions based on over-represented TF binding profiles to find candidate upstream regulators of *RFX* genes and thereby infer the developmental pathways that RFX1–8 are part of. The over-represented TF binding profiles that our analysis uncovered are associated with TFs involved in **(i)** neural development (SP2, ESR1, Creb5, SOX21) [53–56] and neurite outgrowth (KLF16, EGR3) [57, 58], **(ii)** cognitive functions (EGR3, EGR1) [59], **(iii)** craniofacial development (SP8) [60], **(iv)** proliferation of immune cells (EGR3, KLF5) [61, 62], platelet formation (SP3, SP1) [63] and innate immunological memory (ATF7) [64], **(v)** cell cycle control (E2F4) [65, 66] and tumor suppression (ZNF40, MZF1, TFAP2C, JDP2) [67–70], and **(vi)** reproductive functions (ESR1, Tcfl5) [54, 71]. TF binding profiles for RFX TFs themselves were not over-represented, suggesting that autoregulation is not a common feature for the expression of *RFX1–8* genes and that RFX1 autorepression may be the only exception [72].

We validated SP2 and ESR1 by siRNA knockdown and qRT-PCR and found that they act as inhibitors of the *RFX* genes. We assume that these candidate upstream regulators act directly, given the over-representation of their TF binding site profiles in *RFX1–8* promoter and candidate *RFX* enhancer regions. The cellular context we used for experimental validation, human MCF7 breast cancer cells, very likely does not represent all human tissues. Thus, more exact mechanisms of *RFX* gene regulation remain to be analyzed in different cell-type specific environments. At present, there is little evidence for preferences in RFX dimerization patterns [43].

The discovery of new RFX target genes typically starts with searching for X-box motifs, the binding site for RFX TFs. X-box searches have mostly focused on upstream promoter sequences, e.g. upstream of the first exon or of the ATG [23, 27, 73]. Here we expand by relating X-box position to both upstream *and* downstream of human gene TSS locations. X-box position, motif sequence and conservation across species (cf. Henriksson et al. [74]) allow for a precise ranking of hits. With respect to a given gene TSS location we have assigned these hits to a permissive window (− 2300 to + 1400) and a robust window (− 500 to + 500) for the higher ranks. Our data strengthen and surpass previous work in other organisms where functional X-box motifs were found close to the gene start sites [75]. Our type of analysis will enhance the prediction power of future searches for functional X-box motifs, because relating X-box motifs to both upstream *and* downstream of TSS locations of candidate human RFX target genes adds another level of precision to the search procedure. Functional X-box motifs at larger distances from TSS locations (e.g at distal enhancers) are likely to be the exception rather than the norm. The presence of X-box motifs was shown to contribute to the activeness of both promoters and enhancers, whereby distal enhancers that harbor X-box motifs exhibited greater promoter activity than enhancers that lack them [76]. This phenomenon would fit a model where (as found in *Xenopus leavis*) Rfx2 and Foxj1 coordinately regulate ciliary gene expression, with Rfx2 stabilizing Foxj1 binding at chromatin loops [77].

Comparative tissue and cell type specific expression profile clustering represents a complementary approach to X-box searches for the ascertainment of cross-connections between *RFX* genes and candidate sets of downstream target genes. We have used this approach successfully to describe the key roles that defined RFX family members play by regulating only certain target genes in e.g. human testis and the gastrointestinal tract. Combining both methods, X-box searches and expression profile clustering, will be very helpful for the discovery of precise sets of RFX target genes in many different human tissue and cell types.

Studies in mammals suggest that RFX TFs function in terminal cell differentiation or in the maintenance of certain functional specializations. Examples include the differentiation and maintenance of pancreatic β-cells as insulin producers [78], the repression of collagen formation during adult life [79], the maintenance of testis cord integrity [80], the regulation of spermiogenesis and sperm flagellum assembly [17], the maintenance of post-natal auditory hair cells [81], and the regulation of ciliary genes involved in the assembly and maintenance of functional cilia [16]. Interestingly, RFX TFs seem to exert their function on structures connected to polarized cell surfaces, e.g. cilia, immune synapse, neuronal synapse and the vascular face of β-cells [82].

Given such a range of RFX TF functions in different tissue and cell types, elucidating their role in disease will be facilitated when more precise connections can be established between specific RFX protein isoforms, RFX target gene sets and quantity or cell type of expression. So far only *RFX5* and *RFX6* mutations have been linked to defined diseases, while mutations in other *RFX* genes may cause more complex, pleiotropic disease symptoms. Embryonic lethality in *Rfx1*^*−/−*^ mice suggests that Rfx1 function cannot be compensated for [83]. *RFX* mutations may cause ciliopathies, as RFX TFs directly regulate many ciliary genes in different cell and tissue types. The complexity of ciliopathies arises due to primary cilia being present on most human cell types [84]. Very recently, X-box motifs were shown to overlap with type 2 diabetes risk alleles [85], elevating the importance of understanding X-box motif sequence and position, and X-box containing promoter activity in connection to RFX target gene regulation.

Our exhaustive and in-depth characterization of the functional domain composition and the expression profiles of all the eight human *RFX* genes, including upstream regulatory and downstream target gene analysis, in connection with mammalian studies, e.g. investigating *Rfx* mice mutants, will serve as the basis for uncovering and understanding phenotypes or pathologies of *RFX* mutations in humans. For example, one might expect male sterility to be associated with mutations in testis specific *RFX1–4* gene isoforms, or with dys-regulation of testis specific RFX target genes (e.g. *GPR56* [86], *ALMS1* [87] and *RFX1*), or with the role upstream *RFX* regulators (e.g. ESR1 [88, 89]) play in ciliogenesis.

## Conclusions

We provide a comprehensive and systematic characterization of the expression profiles of all the eight human *RFX* genes, including the previously undescribed *RFX8*. We open the window to their potential upstream regulators during development. We advance on how human RFX TFs regulate their target genes. Thereby, our study contributes to the understanding of the different functions for RFX TFs in their specific spatial and temporal context in the different tissue and cell types of humans. Our work will greatly help in uncovering their cell-type specific target gene batteries, essential for elucidating RFX-associated aspects of cellular specialization and terminal functional differentiation. In turn, this will aid in understanding disease mechanisms and outcome.

## Methods

### Extraction and analysis of CAGE TSS locations from the FANTOM5 database

CAGE TSS locations and expression profiles were extracted from FANTOM5 Phase I as downloaded from SSTAR [90]: http://fantom.gsc.riken.jp/5/sstar/Main_Page. FANTOM5 TSS data represent expression profiles from 889 biological samples with assigned detection levels in arbitrary units “tags per million” (TPM) [33]. We categorized all samples into three separate groups: human tissues (135 samples – 80% adult and 20% fetal), human primary cells (170 samples – here represented as the average of the donor replicates) and human cell lines (255 samples), and excluded the time course samples. TSS data were extracted and analyzed, and then named with shorthand p (for promoter) in alphabetical order (pA, pB, pC, etc.) based on the following criteria: (1) if the tissue correlation is equal to or greater than 0.7 and individual TSS locations fall within 100 bp of each other, they were merged into one TSS; (2) if the highest tissue sample TPM is < 1, this TSS was disregarded unless the highest primary cell (in any donor replicate) or cell line TPM is ≥5; (3) the alphabetical order of TSS locations is based on the descending order of its total sum of TPM values in all 889 biological samples after conditions (1) and (2) are met.

A given TSS location is considered as being expressed broadly if it is expressed at TPM > 5 in a large number and variety of tissues (*n* > 10). Conversely, a given TSS location is considered as being expressed specifically if it is expressed at TPM > 5 in a small number of tissues of the same organ (*n* < 10). The exceptions are: **(i)** pA of *RFX4* displays high expression in many tissues (n > 10) but specifically in the brain and spinal cord; **(ii)** *RFX8* TSS locations are either lowly expressed (TPM < 5) or at background noise levels (TPM < 1). Additional information about these and other CAGE TSS locations present in the FANTOM5 database (e.g. the presence or absence of TATA boxes, CpG islands, etc.) has been described by Lizio et al. 2015 [90]. All the genomic coordinates are stated in BED format.

### Transcript validation from novel TSS locations by RT-PCR

A given TSS location was deemed to be a novel transcript isoform for experimental validation when it does not overlap with or is not found within +/− 50 bp of the start site (indicated as exon 1) of known protein-coding transcripts with complete open reading frame description in the Ensembl database (release 81 – July 2015, http://www.ensembl.org/). We designed forward primers to bind either within the novel TSS sequence, or overlapping with the 3′ end of the TSS, or at the most 50 bp downstream from the TSS. Reverse primers were designed to always bind downstream of the ATG, respectively, from the reference Ensembl transcript. In the case of the *RFX8* gene, we designed additional primers that sandwiched the DBD exonic region to confirm the presence or absence of a DBD-encoding exon. We reverse transcribed 1 μg commercial human testis total RNA (Clontech, Cat. No. 636533) and human whole brain total RNA (Clontech, Cat No. 636530) using Invitrogen SuperScript III First-strand Synthesis Super Mix for qRT-PCR (Cat No. 11752–050). We used undiluted cDNA and 40 PCR cycles with the exception of 45 PCR cycles for *RFX8*. 2 μl of the PCR product were cloned using a TOPO TA Cloning Kit (Invitrogen Dual Promoter PCR II-TOPO Vector, Cat No. 450640). Then, 4–10 white colonies from AMP + IPTG/X-gal plates were screened by PCR M13 vector primers, out of which 2–4 independent samples were sequenced with T7 and SP6 universal primers. Sequencing results were analyzed using the BLAT Tool (UCSC Genome Browser, http://genome-euro.ucsc.edu/). In the case of the *RFX8* DBD transcript validation, at least 100 white colonies were screened with PCR M13 vector primers prior to sequencing, given the overall low expression of the *RFX8* gene. Sequences of primers and verified transcripts are listed in Tables S8 and S9, respectively, in Additional file 3.

### Determination of RFX protein domains

Peptide sequences of human RFX1–3 protein domains (AD, DBD, B, C and DIM) as described previously [9, 10] were used to determine the corresponding domains in human RFX4–8 using the T-coffee protein sequence alignment program [91] (http://www.tcoffee.org/). Visualization of the *RFX* transcripts in Fig. 1a with the protein domain composition was done using IBS software [92].

### Positional X-box motif scanning

We scanned for candidate X-box motifs using two known X-box motifs deposited in the JASPAR database [47] (http://jaspar.genereg.net/): human RFX2 (motif MA0600.1, representing a full-site X-box) and RFX5 (motif MA0510.1, representing a half-site X-box). For these scans we used DNA regions of 5000 bp upstream (− 5000) and 2000 bp downstream (+ 2000) as search windows relative to the TSS locations. We selected X-box motifs in the promoter regions, which were captured by the JASPAR built-in scan function (version 5.0_ALPHA) with an 80% threshold. Previously validated X-box motifs were found with these criteria and also independently using the MEME Suite FIMO software (version 4.10.0) [48] (http://meme-suite.org/tools/fimo) with a *p*-value < 0.0001. Positional motif enrichment was ascertained by analyzing in the same way 10 times random TSS sets from all the CAGE TSS locations present in FANTOM5. The graphical smoothing method employed was local polynomial regression fitting (loess) constructed by the *R* package ggplot2::geom_smooth [93] with a confidence interval (C.I.) level = 0.95.

### Multiple TF binding profile analysis for the prediction of candidate RFX regulators

We performed TF binding profile enrichment analyses using the oPOSSUM3 tool [50] with the CORE vertebrate TF binding profiles present in the JASPAR 2016 database [47]. DNA regions of − 5000 to + 2000 bp of the 30 *RFX* TSS locations and − 200 to + 200 bp from the midpoints of 13 candidate *RFX* enhancers were used as search windows (foreground). Candidate *RFX* enhancers were chosen by selecting enhancers present within − 500 kb to + 500 kb of the 30 *RFX* TSS locations, as extracted from Andersson et al. [49] (http://fantom.gsc.riken.jp/5/datafiles/latest/extra/Enhancers/), and whose expressions were significantly correlated (Spearman correlation with multiple testing correction, False Discovery Rate < 0.05) with *RFX* TSS locations based on FANTOM5 CAGE expression values (TPM) in 889 biological samples). As background we considered 10-fold larger sets of DNA regions with %GC matching the ones of the foreground sequences and derived for regions surrounding all phase 1.3 CAGE peak coordinates (http://fantom.gsc.riken.jp/5/datafiles/phase1.3/extra/CAGE_peaks/hg19.cage_peak_coord_permissive.bed.gz; − 5000 bp and + 2000 bp) and phase 2.0 enhancer coordinates (http://fantom.gsc.riken.jp/5/datafiles/phase2.0/extra/Enhancers/human_permissive_enhancers_phase_1_and_2.bed.gz; +/− 200 bp) using BiasAway (https://www.ncbi.nlm.nih.gov/pubmed/24927817). We computed the mean *(m)* and standard deviation *(sd)* of the distribution of all the z-scores (considering the enrichment of the total number of predicted TFBSs) obtained from oPOSSUM3 and put a threshold at *m* + 2 x *sd*.

### Validation of candidate RFX regulators by siRNA knockdown and qRT-PCR

*SP2* and *ESR1* siRNA concentrations (Table S10 in Additional file 3) were optimized for knockdown efficiency (cutoff: more than 2-fold) using qRT-PCR. siRNA and qPCR primer sequences (obtained from Eurofins Genomics: https://www.eurofinsgenomics.eu/) were selected to target all the known protein-coding transcript isoforms. Primer specificities were tested first by common PCR and later by qPCR analyses of the melting curves using two negative controls, a water sample and a cDNA sample without reverse transcriptase. Sequences of qPCR primers with their amplification efficiencies determined in a standard control setup are listed in Table S11 in Additional file 3. The MCF7 breast cancer cell line (Michigan Cancer Foundation) was used as the human cell line listed in the FANTOM5 database as having sufficiently high expression of both the candidate and the *RFX* genes (TPM > 5). MCF7 cells were maintained using DMEM 1 g/L-D-glucose with added pyruvate, 10% FBS, 1% Penicillin/Streptomycin and 1% L-glutamine at 37 °C at 5% CO^2^. Cells were seeded 24 h prior to transfection (150,000 cells in 2 ml in a 6-well plate format). Lipofectamine RNAiMAX (Invitrogen, Cat. No. 13778–030) was mixed with siRNA according to the manufacturer’s instructions. RNA was extracted (RNeasy Mini Kit and DNase Set, QIAGEN) 24 h after transfection and we used 2 biological replicates repeated on three different days of transfection. We converted 1 μg RNA to cDNA (Invitrogen SuperScript III First-strand Synthesis Super Mix for qRT-PCR, Cat No. 11752–050). qPCR was performed for 40 cycles in singleplex technical triplicates using FastStart Universal SYBR Green Master with ROX reference dye (Roche Cat No. 04913914001) on an AB7500 Fast machine. We used 2 μl of 1:3 diluted cDNA from a biological replicate in 10 μl total. Ct levels with automatic threshold were obtained (Additional file 5) and efficiency-adjusted fold-changes were calculated against scrambled (Scr) control siRNA with the geometric mean of *HPRT1* and *HSPCB* as two independent reference genes for normalization [51]. Graph and statistical tests were performed in *R* using the ggplot2 package [93] and two-tailed one-sample Student’s t-test [94].

### siRNA knockdown confirmation by immunoblotting

At 24 h after transfection with siRNAs, MCF7 cells were collected and washed twice with PBS. Whole cell lysates were prepared upon sonicating the cells in RIPA buffer (Sigma-Aldrich, St. Louis, MO, USA) containing PMSF (1 mM, final concentration) and a protease inhibitor cocktail (Sigma-Aldrich, St. Louis, MO, USA). The protein content of these cell lysates was determined using the BCA protein assay kit (Thermo Scientific, Sweden). A total of 40–80 μg of protein was loaded per well, separated on a 12% SDS-PAGE gel (Bio-Rad, Stockholm, Sweden) and transferred to a 0.45 μm pore-sized PVDF membrane (Bio-Rad, Stockholm, Sweden). After transfer, membranes were incubated overnight at 4 °C with primary antibodies (rabbit polyclonal ESR1, Catalog # sc-543, dilution - 1:500, Santa Cruz Biotech; rabbit polyclonal SP2 (A-8), Catalog # sc-17,814, Lot # D0605, dilution - 1:100, Santa Cruz Biotech; rabbit polyclonal beta-tubulin, Catalog # ab6046, dilution - 1:5000, Abcam; mouse monoclonal vinculin, clone V284, Lot # 2627627, dilution - 1:5000, Millipore) diluted in 5% milk. Subsequently, blots were washed and incubated with either horseradish peroxidase-conjugated secondary antibody (polyclonal rabbit anti-mouse/HRP, Lot # 00054403, dilution - 1:3000, Dako Chemicals) or Li-Cor donkey anti-mouse IRDye 800CW (Catalog # 926–32,212, for vinculin) or Li-Cor donkey anti-rabbit IRDye 680LT (Catalog # 926–68,023, for ESR1) for 1 h at room temperature. Imaging was performed using a Li-Cor Odyssey Fc system. An enhanced chemiluminescence technique (WesternBright Sirius ECL substrate, Advansta) was applied for developing the SP2 blot due to low abundance of the target protein. In all other cases, fluorescence signals were acquired. Band intensities were quantified using ImageJ and normalized with the indicated loading controls.

### Human reference sequence

The human reference sequence used is the Human Feb. 2009 (GRCh37/hg19) Assembly.

## Additional files

Additional file 1: Detailed expression values (TPM) for *RFX* TSS locations. Expression values in tags per million (TPM) for all 30 *RFX* TSS locations in all 889 biological samples and their categorization into tissues (135), primary cells (473 donor replicates and 170 merged replicates from the average TPM value of the donor replicates), cell lines (255) and time courses (26). (XLSX 355 kb)

Additional file 2: Hierarchical clustering, expression plots and top 10 tissues, primary cells and cell lines of *RFX* TSS locations. Hierarchical clustering of 30 *RFX* TSS locations (with shorthand p for promoter) based on expression values (TPM) across 135 human tissue samples, using a 1-Pearson correlation distance measure and average linkage method, as computed by the pvclust R package with nboot = 1000 with the numbers representing approximately unbiased (au) *p*-values (Suzuki and Shimodaira, 2006). Tissue clusters are color-coded and represent the groups of tissues with the highest overall expression values: immune system (teal), gastrointestinal tract (purple), testis (green), brain and spinal cord (red), and two minor clusters, uterus and lung (black). *RFX* TSS locations without color code have low expression values (TPM < 5). This is followed by the expression profiles of 30 *RFX* TSS locations in human tissues, primary cells and cell lines, whereby for every one of the eight human *RFX* genes (1–8), summarized TSS profile data are presented vertically (“top-down”), starting with the a tissue plot, followed by a table of the top 10 tissues, a table of the top 10 primary cells and a table of the top 10 cell lines (highest expression levels are listed first, respectively). The tissue plot is the expression level in log (base 10) TPM against tissues that are sorted from the highest to the lowest expressed from 135 tissues, whereby the plot only includes the first 100 tissues. The arbitrary unit for detection of expression is tags per million (TPM) as defined by FANTOM5. We consider TPM < 5 to be lowly expressed and TPM < 1 to be background noise. (PDF 3276 kb)

Additional file 3: Supporting tables, figures and supplementary references. **Table S1.** Summary of *RFX1–8* expression data and novel transcript validation. **Table S2.** Positions of functional domains encoded by *RFX* transcripts. **Table S3.** Experimentally proven, direct RFX target genes in humans from the literature. **Table S4.** Human X-box motifs selected from the JASPAR database. **Table S5.** Experimentally validated human X-box motif sequences in promoter regions that were captured by the scanning criteria. **Table S6.** Experimentally validated human X-box motif sequences that were either in distal regions or that were not captured by the scanning criteria. **Table S7.** *RFX* correlated enhancers within +/− 500 kb of *RFX* TSS locations. **Table S8.** Primer sequences for novel *RFX* transcripts validation. **Table S9.** Verified novel *RFX* transcript sequences. **Table S10.** siRNA sequences for candidate RFX regulators. **Table S11.** qPCR primer sequences and amplification efficiencies for validation of candidate RFX regulators. **Figure S1.** Human RFX1–8 DBD protein sequence alignment. Supplementary references. (DOCX 424 kb)

Additional file 4: Detailed candidate *RFX* regulator oPOSSUM3 scanning results using JASPAR 2016 core vertebrate TF binding profiles. Transcription factor binding sites (TFBS) scanning results from oPOSSUM3 within the promoter and enhancer regions of *RFX1–8* using the CORE vertebrate TF binding profiles in JASPAR 2016. Included are the DNA regions that were considered as foreground and the following TF binding site details: SP2 (specificity protein 2) (JASPAR profile MA0516.1) and ESR1 (estrogen receptor alpha) (MA0112.3). (XLSX 50 kb)

Additional file 5: Ct levels of qRT-PCR, used for validation of candidate *RFX* regulators by siRNA knockdown. Individual Ct levels with automatic threshold obtained on an AB7500 Fast machine for *SP2* and *ESR1* as candidate *RFX* regulators and their respective test siRNA and scrambled (Scr) control siRNA knockdown data on *RFX* genes (*RFX1*, *RFX2*, *RFX3*, *RFX5*, *RFX7*) and the two reference genes (*HPRT1*, *HSPCB*). (XLSX 33 kb)

## Abbreviations

AD: Activating domain
B: B domain
C: C domain
CAGE: Cap Analysis of Gene Expression
DBD: DNA binding domain
DIM: Dimerization domain
FANTOM5: Functional Annotation of Mammalian Genome 5
RFX: Regulatory Factor binding to the X-box
TF: Transcription factor
TFBS: Transcription factor binding site
TPM: Tags per million
TSS: Transcription start site
