# Supplementary material for: Characterization of the human RFX transcription factor family by regulatory and target gene analysis
Source: BMC Genomics. 2018 Mar 6;19:181. doi: 10.1186/s12864-018-4564-6 (PMC5838959; doi:10.1186/s12864-018-4564-6)
Supplement: Supplementary file 2 — Hierarchical clustering, expression plots and top 10 tissues, primary cells and cell lines of RFX TSS locations. Hierarchical clustering of 30 RFX TSS locations (with shorthand p for promoter) based on expression values (TPM) across 135 human tissue samples, using a 1-Pearson correlation distance measure and average linkage method, as computed by the pvclust R package with nboot = 1000 with the numbers representing approximately unbiased (au) p-values (Suzuki and Shimodaira, 2006). Tissue clusters are color-coded and represent the groups of tissues with the highest overall expression values: immune system (teal), gastrointestinal tract (purple), testis (green), brain and spinal cord (red), and two minor clusters, uterus and lung (black). RFX TSS locations without color code have low expression values (TPM < 5). This is followed by the expression profiles of 30 RFX TSS locations in human tissues, primary cells and cell lines, whereby for every one of the eight human RFX genes (1–8), summarized TSS profile data are presented vertically (“top-down”), starting with the a tissue plot, followed by a table of the top 10 tissues, a table of the top 10 primary cells and a table of the top 10 cell lines (highest expression levels are listed first, respectively). The tissue plot is the expression level in log (base 10) TPM against tissues that are sorted from the highest to the lowest expressed from 135 tissues, whereby the plot only includes the first 100 tissues. The arbitrary unit for detection of expression is tags per million (TPM) as defined by FANTOM5. We consider TPM < 5 to be lowly expressed and TPM < 1 to be background noise. (PDF 3276 kb) [file 12864_2018_4564_MOESM2_ESM.pdf]

Characterization of the human RFX transcription factor family by regulatory and target gene analysis

Debora Sugiaman-Trapman, Morana Vitezic, Eeva-Mari Jouhilahti, Anthony Mathelier, Gilbert Lauter, Sougat Misra, Carsten O. Daub, Juha Kere and Peter Swoboda

Additional file 2: Hierarchical clustering, expression plots and top 10 tissues, primary cells and cell lines of *RFX* TSS locations

Hierarchical clustering of 30 *RFX* TSS locations (with shorthand p for promoter) based on expression values (TPM) across 135 human tissue samples, using a 1-Pearson correlation distance measure and average linkage method, as computed by the pvclust *R* package with nboot=1000 with the numbers representing approximately unbiased (au) p-values (Suzuki and Shimodaira, 2006). Tissue clusters are color-coded and represent the groups of tissues with the highest overall expression values: immune system (teal), gastrointestinal tract (purple), testis (green), brain and spinal cord (red), and two minor clusters, uterus and lung (black). *RFX* TSS locations without color code have low expression values (TPM < 5).

This is followed by the expression profiles of 30 *RFX* TSS locations in human tissues, primary cells and cell lines, whereby for every one of the eight human *RFX* genes (1-8), summarized TSS profile data are presented vertically (“top-down”), starting with the a tissue plot, followed by a table of the top 10 tissues, a table of the top 10 primary cells and a table of the top 10 cell lines (highest expression levels are listed first, respectively). The tissue plot is the expression level in log (base 10) TPM against tissues that are sorted from the highest to the lowest expressed from 135 tissues, whereby the plot only includes the first 100 tissues.

The arbitrary unit for detection of expression is tags per million (TPM) as defined by FANTOM5. We consider TPM < 5 to be lowly expressed and TPM < 1 to be background noise.

Suzuki R, Shimodaira H: **Pvclust: an R package for assessing the uncertainty in hierarchical clustering.** *Bioinformatics 2006*, **22**:1540-1542.

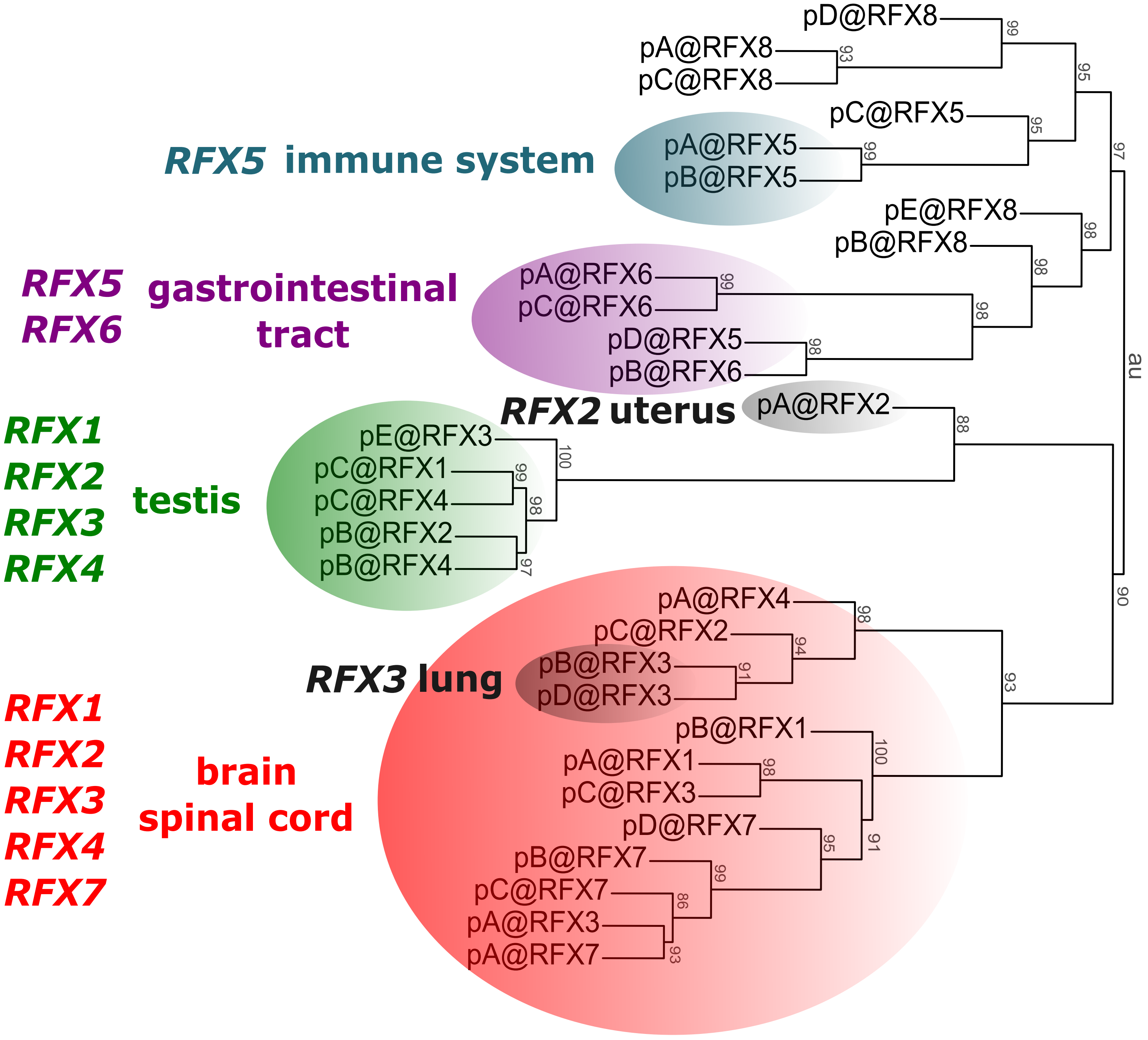

RFX5 immune system
RFX5
RFX6
gastrointestinal tract
RFX2 uterus
RFX1
RFX2
RFX3
RFX4
testis
RFX3 lung
RFX1
RFX2
RFX3
RFX4
RFX7
brain
spinal cord
pD@RFX8
pA@RFX8
pC@RFX8
pC@RFX5
pA@RFX5
pB@RFX5
pE@RFX8
pB@RFX8
pA@RFX6
pC@RFX6
pD@RFX5
pB@RFX6
pA@RFX2
pE@RFX3
pC@RFX1
pC@RFX4
pB@RFX2
pB@RFX4
pA@RFX4
pC@RFX2
pB@RFX3
pD@RFX3
pB@RFX1
pA@RFX1
pC@RFX3
pD@RFX7
pB@RFX7
pC@RFX7
pA@RFX3
pA@RFX7
au

RFX1

pA@RFX1	chr19:14117085..14117141,-
pB@RFX1	chr19:14117148..14117157,-
pC@RFX1	chr19:14117622..14117633,-

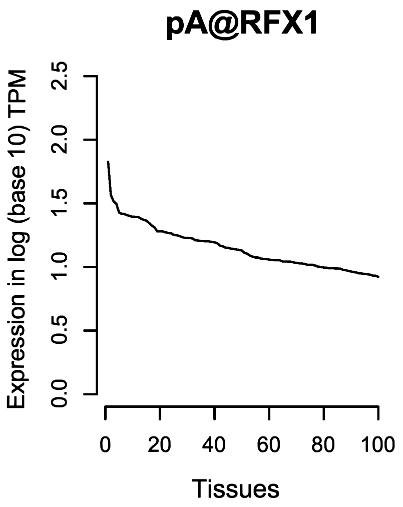

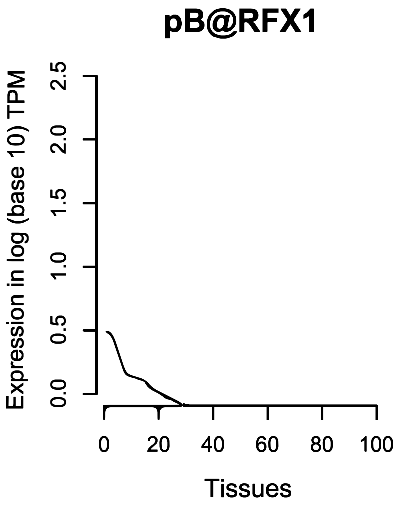

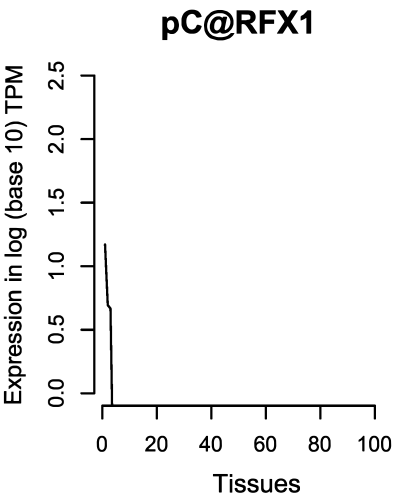

	Top 10 tissues	TPM
1	cerebellum, adult	67
2	occipital cortex, adult	37
3	middle temporal gyrus	33
4	pituitary gland, adult	31
5	parietal lobe, adult	27
6	thymus, fetal	26
7	duodenum, fetal	26
8	spleen, fetal	25
9	medial temporal gyrus, adult	25
10	hippocampus, adult	25

	Top 10 tissues	TPM
1	cerebellum - adult	3.1
2	cerebellum, adult	3.0
3	optic nerve	2.9
4	temporal lobe, fetal	2.5
5	middle temporal gyrus	2.1
6	temporal lobe, fetal	1.8
7	occipital cortex, adult	1.6
8	lung, fetal	1.4
9	submaxillary gland, adult	1.4
10	pineal gland, adult	1.4

	Top 10 tissues	TPM
1	testis, adult	15
2	Clontech Human Universal Reference Total RNA	4.9
3	testis, adult	4.6
4	pituitary gland, adult	0.2
5	placenta, adult	0.1
6	frontal lobe, adult	0.1
7	*all other tissues*	0
8	...	0
9	...	0
10	...	0

	Top 10 primary cells	TPM
1	CD14+ monocytes - mock treated	37
2	Neutrophils	26
3	CD14+ monocytes – treated with Candida	25
4	CD14+ monocytes – treated with B-glucan	23
5	Whole blood (ribopure)	22
6	CD14+ monocytes – treated with Cryptococcus	21
7	Smooth Muscle Cells - Esophageal	21
8	Renal Epithelial Cells	20
9	Pericytes	20
10	Renal Mesangial Cells	19

	Top 10 primary cells	TPM
1	Neutrophils	1.5
2	Smooth Muscle Cells - Esophageal	1.5
3	granulocyte macrophage progenitor	1.3
4	Mesenchymal stem cells - adipose	1.2
5	Hepatocyte	1.2
6	Pericytes	1.1
7	Smooth Muscle Cells - Pulmonary Artery	1.1
8	Renal Glomerular Endothelial Cells	0.9
9	Astrocyte - cerebellum	0.9
10	Fibroblast - Lymphatic	0.8

	Top 10 primary cells	TPM
1	Eosinophils	0.7
2	Basophils	0.5
3	Natural Killer Cells	0.5
4	CD8+ T Cells	0.4
5	CD19+ B Cells	0.4
6	CD4+CD25-CD45RA+ naive conventional T cells	0.4
7	CD14+ monocytes - treated with Candida	0.3
8	CD4+CD25-CD45RA- memory conventional T cells	0.3
9	CD34+ Progenitors	0.3
10	CD4+CD25+CD45RA- memory regulatory T cells	0.3

	Top 10 cell lines	TPM
1	peripheral neuroectodermal tumor cell line:KU-SN	33
2	tridermal teratoma cell line:HGRT	23
3	acute myeloid leukemia (FAB M7) cell line:MKPL-1	23
4	small cell gastrointestinal carcinoma cell line:ECC10	20
5	acute myeloid leukemia (FAB M6) cell line:F-36E	20
6	testicular germ cell embryonal carcinoma cell line:NEC15	18
7	neuroectodermal tumor cell line:FU-RPNT-1	17
8	epitheloid carcinoma cell line: HelaS3 ENCODE	17
9	acute myeloid leukemia (FAB M6) cell line:EEB	16
10	embryonic kidney cell line: HEK293/SLAM infection, 24hr	16

	Top 10 cell lines	TPM
1	testicular germ cell embryonal carcinoma cell line:NEC8	1.8
2	embryonic pancreas cell line:1B2C6	1.3
3	lens epithelial cell line:SRA 01/04	1.2
4	embryonic pancreas cell line:2C6	1.2
5	chronic myelogenous leukemia cell line:K562 ENCODE	1.2
6	hepatoblastoma cell line:HuH-6	1.2
7	bone marrow stromal cell line:StromaNKtert	1.1
8	testicular germ cell embryonal carcinoma cell line	1.1
9	small cell lung carcinoma cell line:DMS 144	1.1
10	rhabdomyosarcoma cell line:RMS-YM	1.0

	Top 10 cell lines	TPM
1	keratoacanthoma cell line:HKA-1	0.3
2	anaplastic large cell lymphoma cell line: Ki-JK	0.3
3	embryonic pancreas cell line:1C3D3	0.3
4	acute myeloid leukemia (FAB M5) cell line:THP-1 (fresh)	0.3
5	leiomyoblastoma cell line: G-402	0.3
6	embryonic kidney cell line: HEK293/SLAM infection, 24hr	0.2
7	melanoma cell line:COLO 679	0.2
8	small cell lung carcinoma cell line:NCI-H82	0.1
9	*all other cell lines*	0
10	...	0

RFX2

pA@RFX2	chr19:6110474..6110551,-
pB@RFX2	chr19:6110555..6110617,-
pC@RFX2	chr19:6110458..6110472,-

pA@RFX2

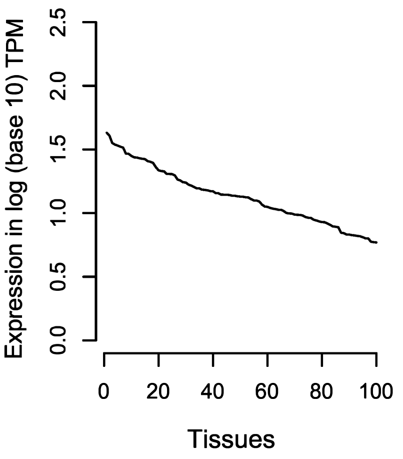

	Top 10 tissues	TPM
1	uterus, adult	43
2	aorta, adult	40
3	smooth muscle, adult	36
4	diaphragm, fetal	35
5	heart, adult, diseased	34
6	heart - mitral valve, adult	33
7	testis, adult	33
8	pituitary gland, adult	29
9	medulla oblongata - adult	29
10	umbilical cord, fetal	28

	Top 10 primary cells	TPM
1	immature langerhans cells	120
2	Mast cell	83
3	Neutrophils	58
4	CD34+ Progenitors	57
5	Eosinophils	51
6	Basophils	46
7	CD14+ monocytes - treated with Group A streptococci	43
8	CD14+ monocytes - mock treated	39
9	CD14+ monocytes - treated with B-glucan	37
10	CD14+ monocytes - treated with Candida	36

	Top 10 cell lines	TPM
1	gastrointestinal carcinoma cell line:ECC12	43
2	extraskeletal myxoid chondrosarcoma cell line:H-EMC-SS	42
3	small-cell gastrointestinal carcinoma cell line:ECC4	32
4	biphenotypic B myelomonocytic leukemia cell line:MV-4-11	22
5	cervical cancer cell line:ME-180	21
6	acute myeloid leukemia (FAB M1) cell line:HYT-1	20
7	acute myeloid leukemia (FAB M0) cell line:Kasumi-3	20
8	mesothelioma cell line:Mero-41	19
9	glioblastoma cell line:A172	19
10	acute myeloid leukemia (FAB M6) cell line	19

pB@RFX2

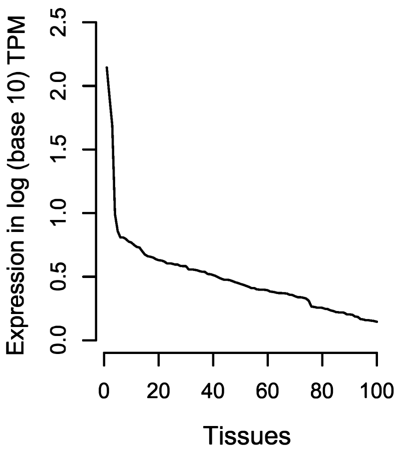

	Top 10 tissues	TPM
1	testis, adult	140
2	testis, adult	81
3	Clontech Human Universal Reference Total RNA	49
4	thymus, fetal	9.8
5	aorta, adult	7.2
6	rectum, fetal	6.4
7	thymus, adult	6.4
8	uterus, adult	6.2
9	dura mater, adult	5.9
10	stomach, fetal	5.8

	Top 10 primary cells	TPM
1	Eosinophils	38
2	Mast cell	26
3	Neutrophils	25
4	immature langerhans cells	24
5	granulocyte macrophage progenitor	17
6	CD14+ Monocytes	15
7	mature adipocyte	15
8	Basophils	14
9	CD14+ monocytes - treated with Candida	12
10	CD34+ Progenitors	12

	Top 10 cell lines	TPM
1	biphenotypic B myelomonocytic leukemia cell line:MV-4-11	12
2	retinoblastoma cell line:Y79	10
3	adrenal cortex adenocarcinoma cell line:SW-13	9.8
4	acute myeloid leukemia (FAB M1) cell line:HYT-1	8.5
5	testicular germ cell embryonal carcinoma cell line:NEC8	8.3
6	bronchioalveolar carcinoma cell line:NCI-H358	8.1
7	acantholytic squamous carcinoma cell line:HCC1806	8.1
8	acute myeloid leukemia (FAB M2) cell line:Kasumi-1	7.8
9	tridermal teratoma cell line:HGRT	7.7
10	anaplastic squamous cell carcinoma cell line:RPMI 2650	7.6

pC@RFX2

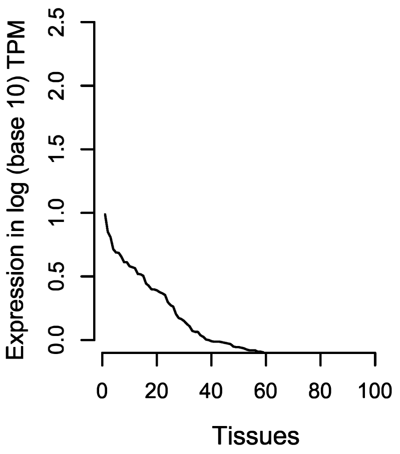

	Top 10 tissues	TPM
1	medulla oblongata - adult	9.8
2	caudate nucleus - adult	7.1
3	trachea, adult	6.5
4	aorta, adult	5.2
5	medulla oblongata, adult	4.9
6	corpus callosum, adult	4.9
7	throat, fetal	4.5
8	lung, fetal	4.1
9	hippocampus, adult	4.1
10	spinal cord, fetal	3.8

	Top 10 primary cells	TPM
1	Mast cell	4.3
2	immature langerhans cells	3.3
3	CD34+ Progenitors	2.8
4	granulocyte macrophage progenitor	2.6
5	salivary acinar cells	1.6
6	Synoviocyte	1.4
7	CD133+ stem cells - adult bone marrow derived	1.2
8	Multipotent Cord Blood Unrestricted Somatic Stem Cells	1.2
9	amniotic membrane cells	1.0
10	Natural Killer Cells	1.0

	Top 10 cell lines	TPM
1	gastrointestinal carcinoma cell line:ECC12	4.6
2	small-cell gastrointestinal carcinoma cell line:ECC4	2.8
3	argyrophil small cell carcinoma cell line:TC-YIK	2.5
4	serous adenocarcinoma cell line:JHOS-2	1.8
5	clear cell carcinoma cell line:TEN	1.6
6	extraskeletal myxoid chondrosarcoma cell line:H-EMC-SS	1.5
7	glioma cell line:GI-1	1.2
8	glioblastoma cell line:A172	1.1
9	mesothelioma cell line:NCI-H2452	1.0
10	acute myeloid leukemia (FAB M1) cell line:HYT-1	0.9

RFX3

pA@RFX3	chr9:3525867..3526016,-
pB@RFX3	chr9:3525727..3525828,-
pC@RFX3	chr9:3526360..3526421,-
pD@RFX3	chr9:3525848..3525863,-
pE@RFX3	chr9:3489406..3489437,-

pA@RFX3

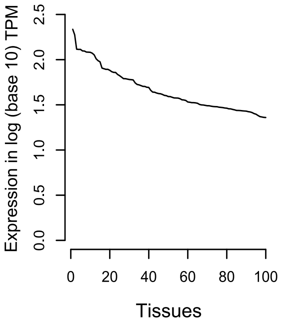

	Top 10 tissues	TPM
1	cerebellum, adult	220
2	parietal lobe, fetal	190
3	parietal lobe, adult	130
4	occipital lobe, fetal	130
5	pineal gland, adult	130
6	temporal lobe, fetal	130
7	pituitary gland, adult	130
8	temporal lobe, fetal	120
9	occipital cortex, adult	120
10	middle temporal gyrus	120

	Top 10 primary cells	TPM
1	Neutrophils	49
2	CD19+ B Cells	44
3	CD4+CD25-CD45RA+ naive conventional T cells	40
4	CD4+CD25+CD45RA+ naive regulatory T cells	38
5	CD14+ monocytes - treated with IFN + N-hexane	34
6	CD4+ T Cells	34
7	Neural stem cells	33
8	CD4+CD25+CD45RA- memory regulatory T cells	33
9	CD4+CD25-CD45RA- memory conventional T cells	32
10	CD14+ monocytes - treated with Trehalose dimycolate (TDM)	31

	Top 10 cell lines	TPM
1	medulloblastoma cell line:D283 Med	110
2	argyrophil small cell carcinoma cell line:TC-YIK	84
3	extraskeletal myxoid chondrosarcoma cell line:H-EMC-SS	68
4	gastrointestinal carcinoma cell line:ECC12	60
5	maxillary sinus tumor cell line:HSQ-89	55
6	small cell gastrointestinal carcinoma cell line:ECC10	55
7	merkel cell carcinoma cell line:MKL-1	54
8	non T non B acute lymphoblastic leukemia (ALL) cell line:P30/OHK	53
9	hepatoblastoma cell line:HuH-6	52
10	cord blood derived cell line:COBL-a 24h infection(-C)	47

pB@RFX3

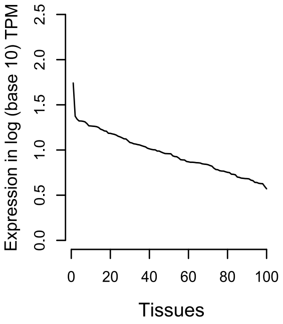

	Top 10 tissues	TPM
1	lung, fetal	55
2	trachea, adult	24
3	occipital lobe, fetal	22
4	amygdala, adult	21
5	temporal lobe, fetal	21
6	spinal cord, adult	21
7	temporal lobe, fetal	21
8	spinal cord - adult	20
9	medulla oblongata, adult	19
10	caudate nucleus, adult	18

	Top 10 primary cells	TPM
1	CD4+CD25-CD45RA- memory conventional T cells	130
2	CD4+ T Cells	120
3	Mast cell	110
4	CD8+ T Cells	96
5	Natural Killer Cells	91
6	CD4+CD25-CD45RA+ naive conventional T cells	89
7	CD4+CD25+CD45RA+ naive regulatory T cells	68
8	Basophils	62
9	CD19+ B Cells	60
10	CD34+ Progenitors	59

	Top 10 cell lines	TPM
1	extraskeletal myxoid chondrosarcoma cell line:H-EMC-SS	37
2	gastrointestinal carcinoma cell line:ECC12	30
3	small-cell gastrointestinal carcinoma cell line:ECC4	23
4	argyrophil small cell carcinoma cell line:TC-YIK	22
5	papillotubular adenocarcinoma cell line:TGBC18TKB	15
6	merkel cell carcinoma cell line:MS-1	13
7	acute myeloid leukemia (FAB M5) cell line:THP-1 (thawed)	12
8	medulloblastoma cell line:D283 Med	11
9	alveolar cell carcinoma cell line:SW 1573	9.7
10	peripheral neuroectodermal tumor cell line:KU-SN	8.6

pC@RFX3

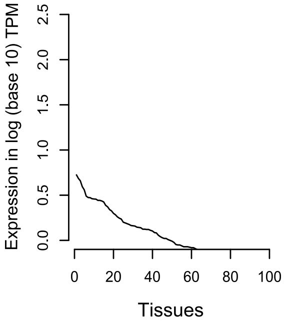

	Top 10 tissues	TPM
1	cerebellum, adult	5.3
2	brain, adult	4.9
3	testis, adult	4.6
4	temporal lobe, fetal	3.9
5	medial temporal gyrus, adult	3.6
6	amygdala, adult	3.1
7	amygdala - adult	3.0
8	spinal cord, adult	3.0
9	parietal lobe, adult	2.9
10	occipital cortex, adult	2.9

	Top 10 primary cells	TPM
1	CD14+ monocytes - treated with Salmonella	20
2	CD14+ monocytes - treated with BCG	18
3	CD14+ monocytes - treated with Group A streptococci	17
4	CD14+ monocytes - treated with lipopolysaccharide	17
5	CD14+ monocytes - treated with Trehalose dimycolate (TDM)	17
6	Natural Killer Cells	14
7	CD133+ stem cells - adult bone marrow derived	12
8	CD4+ T Cells	11
9	CD14+ monocytes - treated with IFN + N-hexane	10
10	CD19+ B Cells	10

	Top 10 cell lines	TPM
1	argyrophil small cell carcinoma cell line:TC-YIK	5.5
2	small cell gastrointestinal carcinoma cell line:ECC10	4.8
3	rectal cancer cell line:TT1TKB	4.8
4	hepatoblastoma cell line:HuH-6	4.7
5	teratocarcinoma cell line:NCR-G1	4.6
6	squamous cell carcinoma cell line:EC-GI-10	4.1
7	normal embryonic palatal mesenchymal cell line:HEPM	3.9
8	mesodermal tumor cell line:HIRS-BM	3.8
9	embryonic kidney cell line: HEK293/SLAM infection, 24hr	3.8
10	medulloblastoma cell line:ONS-76	3.8

pD@RFX3

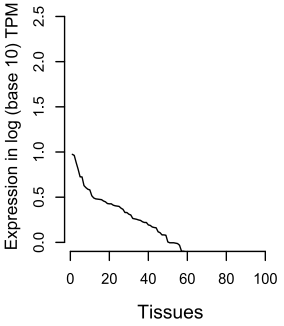

	Top 10 tissues	TPM
1	lung, fetal	9.4
2	medulla oblongata - adult	9.2
3	cerebellum - adult	7.6
4	medulla oblongata, adult	6.4
5	caudate nucleus - adult	5.3
6	spinal cord - adult	5.3
7	occipital cortex - adult	4.3
8	thalamus, adult	4.0
9	middle temporal gyrus	3.9
10	hippocampus, adult	3.8

	Top 10 primary cells	TPM
1	Neutrophils	1.3
2	CD4+CD25+CD45RA+ naive regulatory T cells	1.0
3	Cardiac Myocyte	0.8
4	CD14+ monocytes - treated with Trehalose dimycolate (TDM)	0.8
5	CD4+ T Cells	0.7
6	CD4+CD25+CD45RA- memory regulatory T cells	0.7
7	Natural Killer Cells	0.7
8	CD14+ monocytes - treated with Salmonella	0.7
9	CD8+ T Cells	0.7
10	Mast cell	0.7

	Top 10 cell lines	TPM
1	gastrointestinal carcinoma cell line:ECC12	3.3
2	argyrophil small cell carcinoma cell line:TC-YIK	2.8
3	small-cell gastrointestinal carcinoma cell line:ECC4	1.9
4	carcinosarcoma cell line:JHUCS-1	1.7
5	extraskeletal myxoid chondrosarcoma cell line:H-EMC-SS	1.5
6	peripheral neuroectodermal tumor cell line:KU-SN	1.4
7	acute myeloid leukemia (FAB M6) cell line:F-36E	1.2
8	medulloblastoma cell line:D283 Med	1.1
9	neuroectodermal tumor cell line:FU-RPNT-1	1.1
10	clear cell carcinoma cell line:TEN	1.0

pE@RFX3

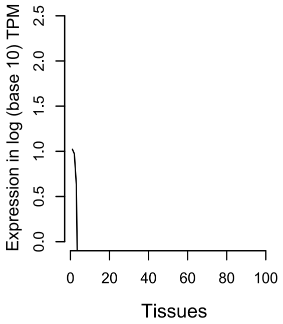

	Top 10 tissues	TPM
1	testis, adult	11
2	testis, adult	9.4
3	Clontech Human Universal Reference Total RNA	4.3
4	cerebellum, adult	0.1
5	brain, adult	0.1
6	*all other tissues*	0
7	...	0
8	...	0
9	...	0
10	...	0

	Top 10 primary cells	TPM
1	CD14+ monocytes - treated with Trehalose dimycolate (TDM)	0.1
2	Mesenchymal stem cells - hepatic	0.1
3	CD4+CD25-CD45RA+ naive conventional T cells	0.1
4	*all other primary cells*	0
5	...	0
6	...	0
7	...	0
8	...	0
9	...	0
10	...	0

	Top 10 cell lines	TPM
1	leukemia, chronic megakaryoblastic cell line:MEG-01	0.1
2	*all other cell lines*	0
3	...	0
4	...	0
5	...	0
6	...	0
7	...	0
8	...	0
9	...	0
10	...	0

RFX4

pA@RFX4	chr12:106976656..106976850,+
pB@RFX4	chr12:107078493..107078533,+
pC@RFX4	chr12:106994905..106994954,+

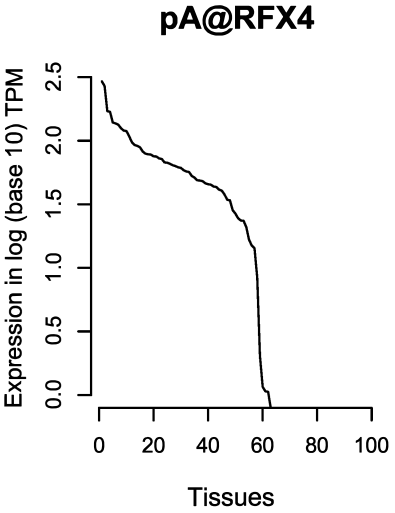

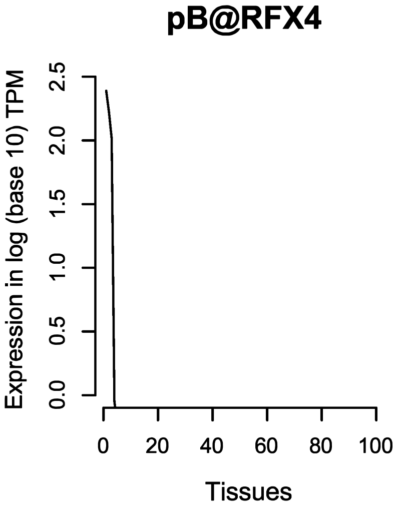

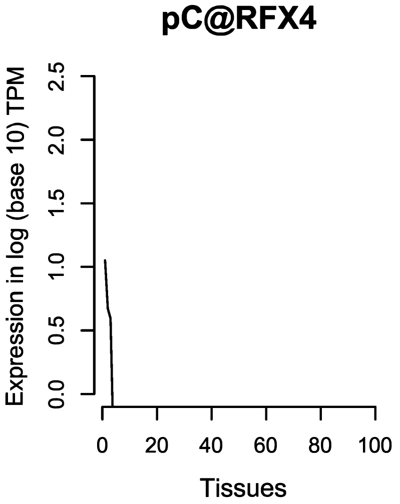

pA@RFX4

	Top 10 tissues	TPM
1	spinal cord - adult	290
2	spinal cord, adult	270
3	thalamus - adult	170
4	medulla oblongata - adult	170
5	caudate nucleus - adult	140
6	locus coeruleus - adult	140
7	medulla oblongata, adult	130
8	globus pallidus - adult	130
9	hippocampus - adult	120
10	spinal cord, fetal	120

	Top 10 primary cells	TPM
1	Neural stem cells	48
2	Neurons	11
3	Astrocyte - cerebral cortex	11
4	Astrocyte - cerebellum	4.8
5	nasal epithelial cells	0.6
6	amniotic membrane cells	0.4
7	Hepatocyte	0.4
8	CD14+ monocytes - treated with lipopolysaccharide	0.2
9	Meningeal Cells	0.1
10	Prostate Epithelial Cells (polarized)	0.1

	Top 10 cell lines	TPM
1	small cell cervical cancer cell line:HCSC-1	57
2	acute myeloid leukemia (FAB M7) cell line:MKPL-1	1.4
3	teratocarcinoma cell line:PA-1	1.3
4	synovial sarcoma cell line:HS-SY-II	0.8
5	testicular germ cell embryonal carcinoma cell line:NEC14	0.5
6	neuroblastoma cell line:NBsusSR	0.5
7	acute myeloid leukemia (FAB M5) cell line:NOMO-1	0.4
8	cord blood derived cell line:COBL-a 24h infection(-C)	0.3
9	neuroblastoma cell line:NB-1	0.2
10	teratocarcinoma cell line:NCC-IT-A3	0.2

pB@RFX4

	Top 10 tissues	TPM
1	testis, adult	240
2	testis, adult	170
3	Clontech Human Universal Reference Total RNA	100
4	caudate nucleus - adult	0.9
5	globus pallidus - adult	0.6
6	thalamus, adult	0.5
7	caudate nucleus, adult	0.5
8	nucleus accumbens, adult	0.5
9	epididymis, adult	0.4
10	SABiosciences XpressRef Human Universal Total RNA	0.2

	Top 10 primary cells	TPM
1	*all primary cells*	0
2	...	0
3	...	0
4	...	0
5	...	0
6	...	0
7	...	0
8	...	0
9	...	0
10	...	0

	Top 10 cell lines	TPM
1	bronchioalveolar carcinoma cell line:NCI-H358	0.3
2	neuroblastoma cell line:NBsusSR	0.2
3	*all other cell lines*	0
4	...	0
5	...	0
6	...	0
7	...	0
8	...	0
9	...	0
10	...	0

pC@RFX4

	Top 10 tissues	TPM
1	testis, adult	11
2	testis, adult	4.7
3	Clontech Human Universal Reference Total RNA	3.9
4	pituitary gland, adult	0.5
5	*all other tissues*	0
6	...	0
7	...	0
8	...	0
9	...	0
10	...	0

	Top 10 primary cells	TPM
1	*all primary cells*	0
2	...	0
3	...	0
4	...	0
5	...	0
6	...	0
7	...	0
8	...	0
9	...	0
10	...	0

	Top 10 cell lines	TPM
1	endometrial carcinoma cell line:OMC-2	1.4
2	neuroblastoma cell line:CHP-134	0.3
3	gastrointestinal carcinoma cell line:ECC12	0.2
4	hepatoma cell line:Li-7	0.1
5	non T non B acute lymphoblastic leukemia (ALL) cell line:P30/OHK	0.1
6	*all other cell lines*	0
7	...	0
8	...	0
9	...	0
10	...	0

RFX5

pA@RFX5	chr1:151319710..151319774,-
pB@RFX5	chr1:151319654..151319698,-
pC@RFX5	chr1:151319318..151319338,-
pD@RFX5	chr1:151319283..151319314,-

pA@RFX5

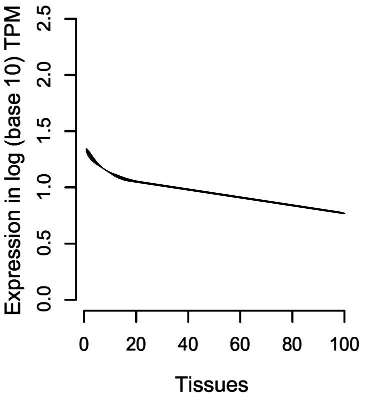

	Top 10 tissues	TPM
1	blood, adult	22
2	tonsil, adult	18
3	appendix, adult	17
4	vein, adult	17
5	thymus, adult	16
6	pons, adult	15
7	thymus, fetal	14
8	spleen, adult	14
9	spleen, fetal	13
10	postcentral gyrus, adult	13

	Top 10 primary cells	TPM
1	CD19+ B Cells (pluriselect)	33
2	CD14+ Monocytes	28
3	CD19+ B Cells	21
4	Dendritic Cells - monocyte immature derived	20
5	CD34+ Progenitors	19
6	Dendritic Cells - plasmacytoid	19
7	CD34+ stem cells - adult bone marrow derived	18
8	Melanocyte - light	18
9	granulocyte macrophage progenitor	18
10	Natural Killer Cells	18

	Top 10 cell lines	TPM
1	transitional-cell carcinoma cell line:JMSU1	44
2	myelodysplastic syndrome cell line:SKM-1	41
3	prostate cancer cell line:DU145	40
4	neuroblastoma cell line:NB-1	37
5	Burkitt's lymphoma cell line:DAUDI	33
6	acute myeloid leukemia (FAB M5) cell line:THP-1 (revived)	33
7	small cell lung carcinoma cell line:LK-2	32
8	acute myeloid leukemia (FAB M5) cell line:THP-1 (fresh)	31
9	acute myeloid leukemia (FAB M5) cell line:THP-1 (thawed)	28
10	lymphoma, malignant, hairy B-cell cell line:MLMA	27

pB@RFX5

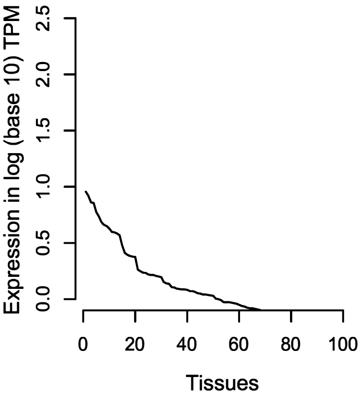

	Top 10 tissues	TPM
1	tonsil, adult	9.0
2	appendix, adult	8.3
3	vein, adult	7.3
4	small intestine, adult	7.2
5	colon, adult	6.0
6	spleen, adult	5.5
7	duodenum, fetal	4.8
8	spleen, fetal	4.6
9	thymus, fetal	4.5
10	thymus, adult	4.3

	Top 10 primary cells	TPM
1	migratory langerhans cells	49
2	CD19+ B Cells (pluriselect)	27
3	CD19+ B Cells	18
4	immature langerhans cells	10
5	Natural Killer Cells	9.9
6	Neutrophils	9.1
7	CD4+CD25+CD45RA- memory regulatory T cells expanded	7.6
8	Eosinophils	7.2
9	Dendritic Cells - monocyte immature derived	6.5
10	CD8+ T Cells	6.3

	Top 10 cell lines	TPM
1	adult T-cell leukemia cell line:ATN-1	60
2	splenic lymphoma with villous lymphocytes cell line:SLVL	28
3	xeroderma pigentosum b cell line:XPL 17	20
4	b cell line:RPMI1788	19
5	B lymphoblastoid cell line: GM12878 ENCODE	19
6	lymphoma, malignant, hairy B-cell cell line:MLMA	18
7	B lymphoblastoid cell line: GM12878 ENCODE	17
8	B lymphoblastoid cell line: GM12878 ENCODE	16
9	acute lymphoblastic leukemia (B-ALL) cell line:BALL-1	14
10	plasma cell leukemia cell line:ARH-77	13

pC@RFX5

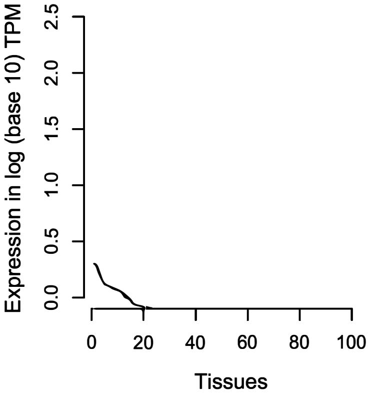

	Top 10 tissues	TPM
1	brain, fetal	2.0
2	thymus, adult	1.9
3	pineal gland - adult	1.6
4	thymus, fetal	1.4
5	temporal lobe, fetal	1.3
6	spleen, fetal	1.3
7	spinal cord, fetal	1.3
8	thyroid, fetal	1.2
9	cerebellum, adult	1.2
10	skeletal muscle, fetal	1.2

	Top 10 primary cells	TPM
1	CD34+ Progenitors	5.4
2	Dendritic Cells - plasmacytoid	4.3
3	migratory langerhans cells	3.5
4	CD133+ stem cells - adult bone marrow derived	2.9
5	CD34+ stem cells - adult bone marrow derived	2.9
6	CD19+ B Cells	2.0
7	Mast cell - stimulated	2.0
8	CD19+ B Cells (pluriselect)	1.7
9	CD14+ Monocytes	1.5
10	Ciliary Epithelial Cells	1.4

	Top 10 cell lines	TPM
1	neuroectodermal tumor cell line:FU-RPNT-1	6.2
2	cord blood derived cell line:COBL-a 24h infection	4.7
3	neuroblastoma cell line:NB-1	4.4
4	cord blood derived cell line:COBL-a untreated	3.9
5	adult T-cell leukemia cell line:ATN-1	3.7
6	cord blood derived cell line:COBL-a 24h infection(-C)	3.6
7	Burkitt's lymphoma cell line:RAJI	3.0
8	acute lymphoblastic leukemia (B-ALL) cell line:BALL-1	3.0
9	hairy cell leukemia cell line:Mo	2.9
10	anaplastic squamous cell carcinoma cell line:RPMI 2650	2.7

pD@RFX5

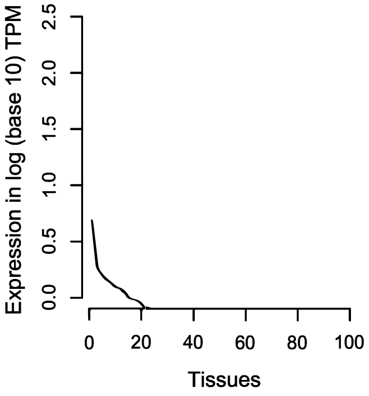

	Top 10 tissues	TPM
1	duodenum, fetal	4.8
2	parietal lobe, fetal	3.3
3	temporal lobe, fetal	1.8
4	spinal cord, fetal	1.7
5	duodenum, fetal	1.6
6	skin, fetal	1.5
7	occipital cortex - adult	1.4
8	appendix, adult	1.4
9	temporal lobe, fetal	1.3
10	uterus, fetal	1.2

	Top 10 primary cells	TPM
1	CD133+ stem cells - adult bone marrow derived	7.3
2	CD34+ Progenitors	5.9
3	migratory langerhans cells	5.2
4	Eosinophils	4.6
5	Neutrophils	4.2
6	immature langerhans cells	4.1
7	Mast cell - stimulated	3.5
8	CD19+ B Cells	3.3
9	CD14+ Monocytes	3.2
10	CD14+ monocytes - treated with Candida	3.1

	Top 10 cell lines	TPM
1	cord blood derived cell line:COBL-a 24h infection(-C)	7.4
2	cord blood derived cell line:COBL-a untreated	4.9
3	cord blood derived cell line:COBL-a 24h infection	4.7
4	medulloblastoma cell line:D283 Med	3.3
5	plasma cell leukemia cell line:ARH-77	3.1
6	mycosis fungoides, T cell lymphoma cell line:HuT 102 TIB-162	3.0
7	acute myeloid leukemia (FAB M1) cell line:HYT-1	3.0
8	hairy cell leukemia cell line:Mo	2.6
9	neuroectodermal tumor cell line:FU-RPNT-2	2.4
10	neuroectodermal tumor cell line:FU-RPNT-1	2.3

RFX6

pA@RFX6	chr6:117198400..117198441,+
pB@RFX6	chr6:117198376..117198383,+
pC@RFX6	chr6:117198458..117198464,+

pA@RFX6

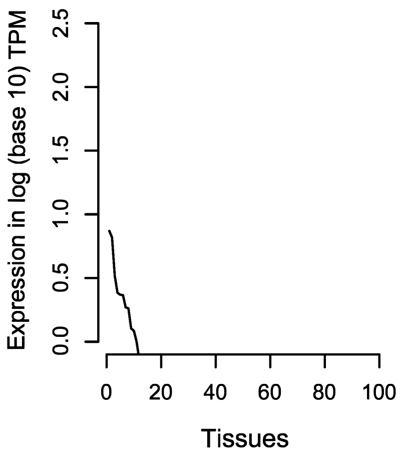

pB@RFX6

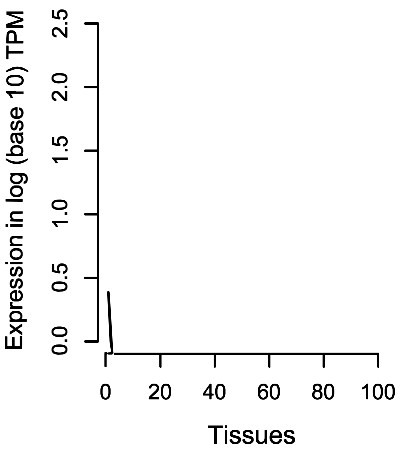

pC@RFX6

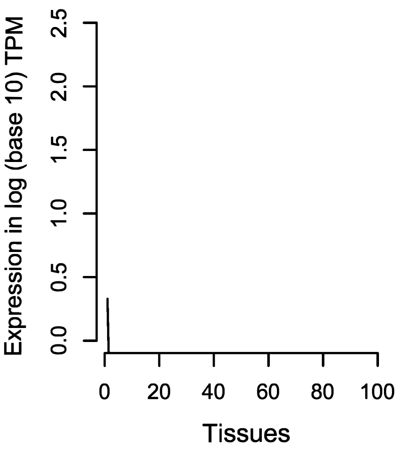

	Top 10 tissues	TPM
1	duodenum, fetal	7.4
2	stomach, fetal	6.6
3	small intestine, fetal	3.3
4	duodenum, fetal	2.4
5	pancreas, adult	2.3
6	small intestine, adult	2.3
7	colon, adult	1.9
8	rectum, fetal	1.8
9	seminal vesicle, adult	1.3
10	colon, fetal	1.2

	Top 10 tissues	TPM
1	duodenum, fetal	2.4
2	rectum, fetal	0.9
3	breast, adult	0.7
4	pituitary gland - adult	0.6
5	duodenum, fetal	0.5
6	pituitary gland, adult	0.2
7	small intestine, fetal	0.2
8	colon, adult	0.2
9	placenta, adult	0.1
10	small intestine, adult	0.1

	Top 10 tissues	TPM
1	duodenum, fetal	2.1
2	small intestine, adult	0.2
3	colon, adult	0.2
4	testis, adult	0.1
5	*all other tissues*	0
6	...	0
7	...	0
8	...	0
9	...	0
10	...	0

	Top 10 primary cells	TPM
1	Prostate Epithelial Cells (polarized)	0.4
2	Intestinal epithelial cells (polarized)	0.3
3	*all other primary cells*	0
4	...	0
5	...	0
6	...	0
7	...	0
8	...	0
9	...	0
10	...	0

	Top 10 primary cells	TPM
1	Prostate Stromal Cells	0.1
2	*all other primary cells*	0
3	...	0
4	...	0
5	...	0
6	...	0
7	...	0
8	...	0
9	...	0
10	...	0

	Top 10 primary cells	TPM
1	*all primary cells*	0
2	...	0
3	...	0
4	...	0
5	...	0
6	...	0
7	...	0
8	...	0
9	...	0
10	...	0

	Top 10 cell lines	TPM
1	argyrophil small cell carcinoma cell line:TC-YIK	130
2	gastrointestinal carcinoma cell line:ECC12	85
3	embryonic kidney cell line: HEK293/SLAM untreated	0.8
4	embryonic kidney cell line: HEK293/SLAM infection, 24hr	0.5
5	hepatoma cell line:Li-7	0.3
6	prostate cancer cell line:PC-3	0.2
7	gall bladder carcinoma cell line:TGBC14TKB	0.2
8	colon carcinoma cell line:CACO-2	0.1
9	liposarcoma cell line:SW 872	0.1
10	*all other cell lines*	0

	Top 10 cell lines	TPM
1	argyrophil small cell carcinoma cell line:TC-YIK	9.6
2	gastrointestinal carcinoma cell line:ECC12	2.6
3	embryonic kidney cell line: HEK293/SLAM untreated	0.2
4	gall bladder carcinoma cell line:TGBC14TKB	0.2
5	liposarcoma cell line:SW 872	0.1
6	testicular germ cell embryonal carcinoma cell line:ITO-II	0.1
7	*all other cell lines*	0
8	...	0
9	...	0
10	...	0

	Top 10 cell lines	TPM
1	argyrophil small cell carcinoma cell line:TC-YIK	7.1
2	gastrointestinal carcinoma cell line:ECC12	3.6
3	*all other cell lines*	0
4	...	0
5	...	0
6	...	0
7	...	0
8	...	0
9	...	0
10	...	0

RFX7

pA@RFX7	chr15:56535946..56535987,-
pB@RFX7	chr15:56535468..56535521,-
pC@RFX7	chr15:56535722..56535764,-
pD@RFX7	chr15:56535922..56535936,-

pA@RFX7

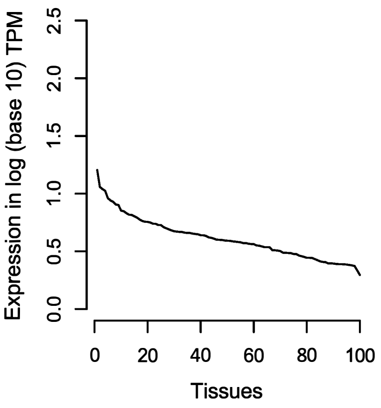

	Top 10 tissues	TPM
1	cerebellum, adult	16
2	pineal gland, adult	11
3	parietal lobe, fetal	11
4	occipital lobe, fetal	11
5	cerebellum - adult	9.2
6	temporal lobe, fetal	8.7
7	occipital cortex - adult	8.5
8	pineal gland - adult	8.0
9	brain, fetal	8.0
10	skeletal muscle - soleus muscle	7.1

	Top 10 primary cells	TPM
1	Neural stem cells	9.0
2	CD34+ Progenitors	5.5
3	CD8+ T Cells	4.0
4	CD4+CD25-CD45RA- memory conventional T cells expanded	3.7
5	salivary acinar cells	3.5
6	Natural Killer Cells	3.5
7	Sertoli Cells	3.2
8	Chondrocyte - re diff	3.1
9	CD4+CD25+CD45RA- memory regulatory T cells	3.1
10	Astrocyte - cerebral cortex	3.0

	Top 10 cell lines	TPM
1	breast carcinoma cell line:MDA-MB-453	10
2	small cell gastrointestinal carcinoma cell line:ECC10	8.6
3	anaplastic squamous cell carcinoma cell line:RPMI 2650	6.5
4	mesodermal tumor cell line:HIRS-BM	6.3
5	lymphoma, malignant, hairy B-cell cell line:MLMA	6.2
6	neuroblastoma cell line:CHP-134	5.6
7	bone marrow stromal cell line:StromaNKtert	5.6
8	neuroblastoma cell line:NH-12	5.4
9	carcinoid cell line:SK-PN-DW	5.3
10	somatostatinoma cell line:QGP-1	5.0

pB@RFX7

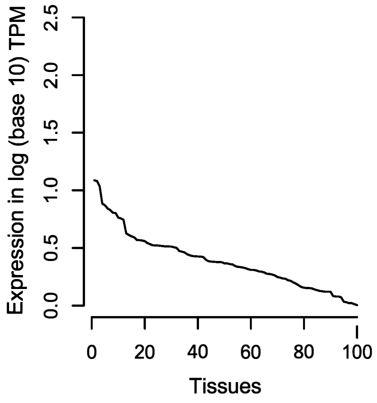

	Top 10 tissues	TPM
1	cerebellum - adult	12
2	parietal lobe, fetal	12
3	occipital lobe, fetal	11
4	temporal lobe, fetal	7.7
5	temporal lobe, fetal	7.4
6	pineal gland - adult	7.0
7	cerebellum, adult	6.8
8	rectum, fetal	6.4
9	occipital lobe, adult	6.4
10	skeletal muscle, fetal	5.8

	Top 10 primary cells	TPM
1	granulocyte macrophage progenitor	17
2	CD34+ Progenitors	12
3	CD133+ stem cells - adult bone marrow derived	8.2
4	Neural stem cells	7.9
5	CD34+ stem cells - adult bone marrow derived	7.6
6	Mast cell - stimulated	6.9
7	Mast cell	4.7
8	salivary acinar cells	3.4
9	Neurons	3.3
10	CD14+monocytes - treated with B-glucan	2.4

	Top 10 cell lines	TPM
1	extraskeletal myxoid chondrosarcoma cell line:H-EMC-SS	20
2	acute myeloid leukemia (FAB M7) cell line:MKPL-1	14
3	Hodgkin's lymphoma cell line:HD-Mar2	11
4	neuroblastoma cell line:CHP-134	11
5	acute myeloid leukemia (FAB M4) cell line:FKH-1	10
6	retinoblastoma cell line:Y79	8.9
7	acute myeloid leukemia (FAB M6) cell line:F-36E	8.6
8	small cell lung carcinoma cell line:NCI-H82	8.0
9	mycosis fungoides, T cell lymphoma cell line:HuT 102 TIB-162	7.0
10	neuroblastoma cell line:NH-12	6.8

pC@RFX7

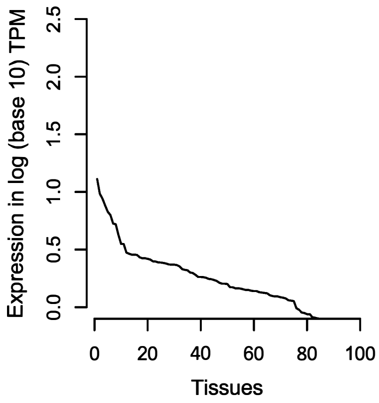

	Top 10 tissues	TPM
1	cerebellum, adult	13
2	pineal gland - adult	9.6
3	parietal lobe, fetal	8.8
4	cerebellum - adult	7.6
5	pineal gland, adult	6.7
6	occipital lobe, fetal	6.3
7	cerebellum, adult	5.3
8	temporal lobe, fetal	5.2
9	occipital cortex - adult	4.3
10	thymus, adult	3.6

	Top 10 primary cells	TPM
1	Mast cell - stimulated	7.4
2	Neural stem cells	2.8
3	Fibroblast - Choroid Plexus	2.8
4	CD34+ Progenitors	2.7
5	Hepatic Sinusoidal Endothelial Cells	1.8
6	Pericytes	1.6
7	Neurons	1.6
8	salivary acinar cells	1.4
9	CD4+CD25+CD45RA+ naive regulatory T cells	1.3
10	granulocyte macrophage progenitor	1.3

	Top 10 cell lines	TPM
1	acute myeloid leukemia (FAB M7) cell line:MKPL-1	8.5
2	acute myeloid leukemia (FAB M6) cell line:F-36E	6.2
3	neuroepithelioma cell line:SK-N-MC	6.2
4	retinoblastoma cell line:Y79	6.0
5	Wilms' tumor cell line:HFWT	4.8
6	sacrococcigeal teratoma cell line:HTST	4.8
7	extraskeletal myxoid chondrosarcoma cell line:H-EMC-SS	4.3
8	myxofibrosarcoma cell line:MFH-ino	4.0
9	tridermal teratoma cell line:HGRT	3.9
10	non T non B acute lymphoblastic leukemia (ALL) cell line:P30/OHK	3.6

pD@RFX7

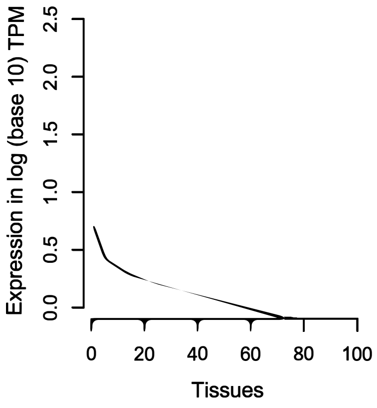

	Top 10 tissues	TPM
1	cerebellum, adult	5.0
2	occipital lobe, fetal	4.0
3	skeletal muscle, adult	3.8
4	pituitary gland, adult	3.4
5	cerebellum, adult	2.7
6	occipital cortex, adult	2.6
7	spinal cord, fetal	2.5
8	umbilical cord, fetal	2.4
9	parietal lobe - adult	2.3
10	parietal lobe, fetal	2.2

	Top 10 primary cells	TPM
1	salivary acinar cells	2.8
2	CD34+ Progenitors	1.9
3	CD8+ T Cells	1.7
4	granulocyte macrophage progenitor	1.3
5	Natural Killer Cells	1.2
6	CD34+ stem cells - adult bone marrow derived	1.1
7	Mast cell	1.0
8	Astrocyte - cerebral cortex	0.9
9	Adipocyte - omental	0.9
10	Neural stem cells	0.9

	Top 10 cell lines	TPM
1	breast carcinoma cell line:MDA-MB-453	7.4
2	acute myeloid leukemia (FAB M4) cell line:FKH-1	5.0
3	lung adenocarcinoma cell line:PC-14.CNhs10726.10408-106B3	4.2
4	extraskeletal myxoid chondrosarcoma cell line:H-EMC-SS	3.8
5	small cell gastrointestinal carcinoma cell line:ECC10	3.6
6	bone marrow stromal cell line:StromaNKtert	3.3
7	mesothelioma cell line:ACC-MESO-4	2.6
8	mesodermal tumor cell line:HIRS-BM	2.5
9	anaplastic squamous cell carcinoma cell line:RPMI 2650	2.3
10	osteoclastoma cell line:Hs 706.T	2.3

RFX8

pA@RFX8	chr2:102091566..102091581,-
pB@RFX8	chr2:102091144..102091183,-
pC@RFX8	chr2:102091478..102091490,-
pD@RFX8	chr2:102091590..102091601,-
pE@RFX8	chr2:102091514..102091528,-

pA@RFX8

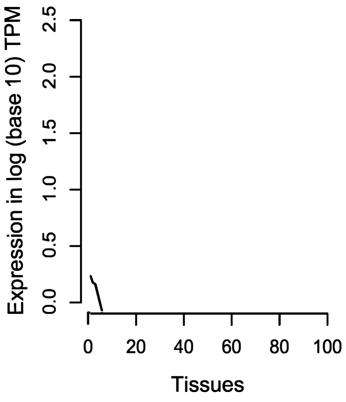

	Top 10 tissues	TPM
1	thymus, adult	1.7
2	breast, adult	1.5
3	heart - tricuspid valve	1.4
4	thymus, fetal	1.1
5	brain, adult	1.0
6	pancreas, adult	0.8
7	trachea, adult	0.7
8	heart, fetal	0.6
9	lymph node, adult	0.5
10	skin, fetal	0.5

	Top 10 primary cells	TPM
1	Fibroblast - Dermal	12
2	granulocyte macrophage progenitor	10
3	CD14+ monocytes - treated with B-glucan	9.5
4	CD14+ monocytes - treated with Salmonella	8.6
5	Chondrocyte - re diff	8.6
6	Preadipocyte - visceral	8.3
7	mesenchymal precursor cell - ovarian cancer left ovary	7.3
8	Fibroblast - Periodontal Ligament	6.9
9	Preadipocyte - subcutaneous	6.9
10	Pancreatic stromal cells	6.9

	Top 10 cell lines	TPM
1	schwannoma cell line:HS-PSS	31
2	schwannoma cell line:HS-PSS	30
3	Hodgkin's lymphoma cell line:HD-Mar2	26
4	biphenotypic B myelomonocytic leukemia cell line:MV-4-11	18
5	myxofibrosarcoma cell line:NMFH-1	17
6	acute myeloid leukemia (FAB M5) cell line:P31/FUJ	12
7	osteosarcoma cell line:143B/TK^(-)neo^(R)	9.7
8	fibrosarcoma cell line:HT-1080	9.0
9	acute myeloid leukemia (FAB M5) cell line:NOMO-1	8.9
10	acute myeloid leukemia (FAB M4eo) cell line:EoL-1	8.8

pB@RFX8

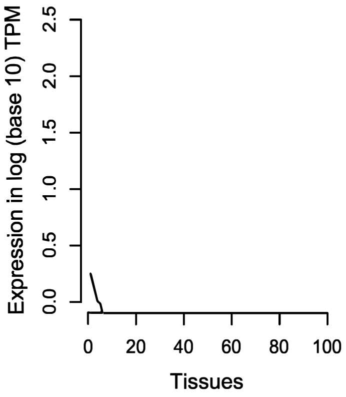

	Top 10 tissues	TPM
1	medial frontal gyrus - adult	1.8
2	penis, adult	1.5
3	duodenum, fetal	1.2
4	amygdala - adult	1.0
5	skin, fetal	1.0
6	parietal lobe - adult	0.8
7	trachea, fetal	0.7
8	vagina, adult	0.7
9	cerebral meninges, adult	0.7
10	medial temporal gyrus - adult	0.6

	Top 10 primary cells	TPM
1	Preadipocyte - visceral	13
2	Smooth Muscle Cells - Internal Thoracic Artery	8.6
3	Preadipocyte - subcutaneous	8.4
4	mesenchymal precursor cell - adipose	6.6
5	tenocyte	5.6
6	mesenchymal precursor cell - cardiac	5.5
7	mesenchymal precursor cell - ovarian cancer left ovary	5.2
8	Fibroblast - Gingival	4.4
9	Smooth Muscle Cells - Uterine	4.3
10	mesenchymal precursor cell - bone marrow	4.1

	Top 10 cell lines	TPM
1	schwannoma cell line:HS-PSS	39
2	schwannoma cell line:HS-PSS	38
3	epithelioid sarcoma cell line:HS-ES-1	27
4	peripheral neuroectodermal tumor cell line:KU-SN	11
5	fibrosarcoma cell line:HT-1080	6.3
6	myxofibrosarcoma cell line:NMFH-1	5.9
7	mesothelioma cell line:NCI-H2052	5.7
8	Hodgkin's lymphoma cell line:HD-Mar2	5.3
9	alveolar cell carcinoma cell line:SW 1573	4.8
10	acute lymphoblastic leukemia (T-ALL) cell line:Jurkat	4.3

pC@RFX8

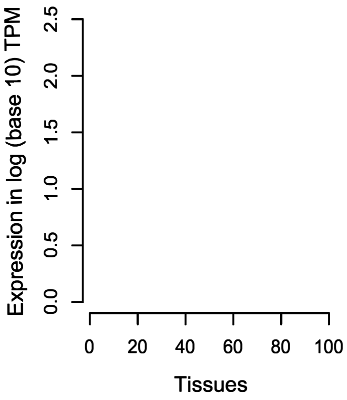

	Top 10 tissues	TPM
1	heart - tricuspid valve, adult	0.7
2	placenta, adult	0.3
3	vein, adult	0.2
4	thyroid, fetal	0.2
5	heart, adult, diseased post-infarction	0.2
6	thymus, adult	0.2
7	locus coeruleus - adult	0.1
8	caudate nucleus, adult	0.1
9	trachea, adult	0.1
10	parietal lobe, adult	0.1

	Top 10 primary cells	TPM
1	Chondrocyte - re diff	5.0
2	Preadipocyte - visceral	4.3
3	granulocyte macrophage progenitor	3.8
4	Smooth Muscle Cells - Aortic	3.7
5	immature langerhans cells	3.4
6	Preadipocyte - subcutaneous	3.1
7	Fibroblast - Dermal	2.8
8	mesenchymal precursor cell - ovarian cancer left ovary	2.6
9	Smooth Muscle Cells - Pulmonary Artery	2.3
10	mesenchymal precursor cell - adipose	2.0

	Top 10 cell lines	TPM
1	schwannoma cell line:HS-PSS	5.3
2	schwannoma cell line:HS-PSS	5.1
3	osteosarcoma cell line:143B/TK^(-)neo^(R)	3.5
4	epithelioid sarcoma cell line:HS-ES-1	3.4
5	biphenotypic B myelomonocytic leukemia cell line:MV-4-11	1.5
6	acute lymphoblastic leukemia (T-ALL) cell line:Jurkat	1.4
7	fibrosarcoma cell line:HT-1080	1.1
8	acute myeloid leukemia (FAB M4eo) cell line:EoL-3	1.0
9	chronic myeloblastic leukemia (CML) cell line:KCL-22	0.9
10	myxofibrosarcoma cell line:NMFH-1	0.9

pD@RFX8

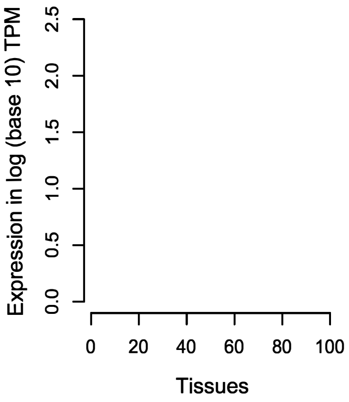

	Top 10 tissues	TPM
1	breast, adult	0.7
2	spinal cord, fetal	0.4
3	uterus, fetal	0.4
4	trachea, adult	0.1
5	thymus, adult	0.1
6	pons, adult	0.1
7	postcentral gyrus, adult	0.1
8	occipital pole, adult	0.1
9	temporal lobe, adult	0.1
10	parietal lobe, adult	0.1

	Top 10 primary cells	TPM
1	mesenchymal precursor cell - adipose	2.8
2	granulocyte macrophage progenitor	2.6
3	Smooth Muscle Cells - Pulmonary Artery	2.3
4	Fibroblast - Dermal	2.1
5	Chondrocyte - re diff	1.7
6	Smooth Muscle Cells - Carotid	1.5
7	Preadipocyte - visceral	1.5
8	Skeletal Muscle Cells	1.4
9	Preadipocyte - subcutaneous	1.3
10	mesenchymal precursor cell - cardiac	1.1

	Top 10 cell lines	TPM
1	schwannoma cell line:HS-PSS	6.7
2	schwannoma cell line:HS-PSS	4.7
3	Hodgkin's lymphoma cell line:HD-Mar2	4.4
4	myxofibrosarcoma cell line:NMFH-1	2.0
5	acute myeloid leukemia (FAB M5) cell line:P31/FUJ	1.7
6	acute myeloid leukemia (FAB M4eo) cell line:EoL-1	1.5
7	fibrosarcoma cell line:HT-1080	1.0
8	epithelioid sarcoma cell line:HS-ES-1	0.9
9	acute myeloid leukemia (FAB M5) cell line:NOMO-1	0.9
10	mesothelioma cell line:NCI-H2052	0.7

pE@RFX8

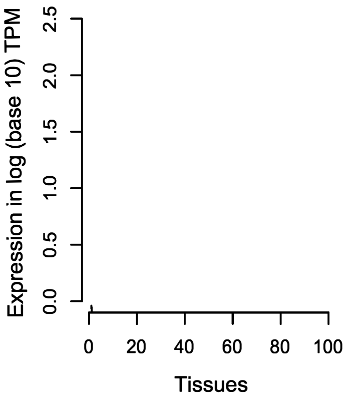

	Top 10 tissues	TPM
1	rectum, fetal	0.9
2	temporal lobe, fetal	0.3
3	heart - mitral valve	0.3
4	middle temporal gyrus	0.1
5	paracentral gyrus, adult	0.1
6	locus coeruleus - adult	0.1
7	parietal lobe, adult	0.1
8	thymus, fetal	0.1
9	kidney, adult	0.1
10	temporal lobe, adult	0.1

	Top 10 primary cells	TPM
1	granulocyte macrophage progenitor	3.8
2	Preadipocyte - visceral	2.0
3	CD14+ monocytes - treated with B-glucan	1.9
4	immature langerhans cells	1.8
5	Preadipocyte - subcutaneous	1.7
6	Chondrocyte - re diff	1.7
7	Fibroblast - Dermal	1.5
8	Mesenchymal stem cells - adipose	1.3
9	Skeletal Muscle Cells	1.1
10	CD14+ monocytes - treated with Candida	1.0

	Top 10 cell lines	TPM
1	schwannoma cell line:HS-PSS	4.3
2	biphenotypic B myelomonocytic leukemia cell line:MV-4-11	2.6
3	acute myeloid leukemia (FAB M4eo) cell line:EoL-1	2.3
4	acute myeloid leukemia (FAB M5) cell line:U-937 DE-4	1.8
5	acute myeloid leukemia (FAB M2) cell line:Kasumi-6	1.7
6	acute myeloid leukemia (FAB M4eo) cell line:EoL-3	1.6
7	schwannoma cell line:HS-PSS, tech_rep2	1.5
8	myxofibrosarcoma cell line:NMFH-1	1.5
9	osteosarcoma cell line:143B/TK^(-)neo^(R)	1.4
10	Hodgkin's lymphoma cell line:HD-Mar2	1.3
