# Supplementary material for: Characterization of the human RFX transcription factor family by regulatory and target gene analysis
Source: BMC Genomics. 2018 Mar 6;19:181. doi: 10.1186/s12864-018-4564-6 (PMC5838959; doi:10.1186/s12864-018-4564-6)
Supplement: Supplementary file 3 — Supporting tables, figures and supplementary references. Table S1. Summary of RFX1–8 expression data and novel transcript validation. Table S2. Positions of functional domains encoded by RFX transcripts. Table S3. Experimentally proven, direct RFX target genes in humans from the literature. Table S4. Human X-box motifs selected from the JASPAR database. Table S5. Experimentally validated human X-box motif sequences in promoter regions that were captured by the scanning criteria. Table S6. Experimentally validated human X-box motif sequences that were either in distal regions or that were not captured by the scanning criteria. Table S7. RFX correlated enhancers within +/− 500 kb of RFX TSS locations. Table S8. Primer sequences for novel RFX transcripts validation. Table S9. Verified novel RFX transcript sequences. Table S10. siRNA sequences for candidate RFX regulators. Table S11. qPCR primer sequences and amplification efficiencies for validation of candidate RFX regulators. Figure S1. Human RFX1–8 DBD protein sequence alignment. Supplementary references. (DOCX 424 kb) [file 12864_2018_4564_MOESM3_ESM.docx]

**Characterization of the human RFX transcription factor family by regulatory and target gene analysis**

Debora Sugiaman-Trapman, Morana Vitezic, Eeva-Mari Jouhilahti, Anthony Mathelier, Gilbert Lauter, Sougat Misra, Carsten O. Daub, Juha Kere and Peter Swoboda

**Additional file 3: Supporting tables, figures and supplementary references**

Table S1: Summary of *RFX1-8* expression data and novel transcript validation

Table S2 Positions of functional domains encoded by *RFX* transcripts

Table S3: Experimentally proven, direct RFX target genes in humans from the literature

Table S4: Human X-box motifs selected from the JASPAR database

Table S5: Experimentally validated human X-box motif sequences in promoter regions that were captured by the scanning criteria

Table S6: Experimentally validated human X-box motif sequences that were either in distal regions or that were not captured by the scanning criteria

Table S7: *RFX* correlated enhancers within +/- 500 kb of *RFX* TSS locations

Table S8: Primer sequences for novel *RFX* transcripts validation

Table S9: Verified novel *RFX* transcript sequences

Table S10: siRNA sequences for candidate RFX regulators

Table S11: qPCR primer sequences and amplification efficiencies for validation of candidate RFX regulators

Figure S1: Human RFX1-8 DBD protein sequence alignment

Supplementary references

**Table S1: Summary of *RFX1-8* expression data and novel transcript validation**

| **Gene name** | **TSS** | **TSS location** | **Tissue profile summary** | | **Ensembl transcript ID**  **(RefSeq transcript ID)** | **Experimental validation of novel TSS locations** | **Ensembl protein ID**  **(Refseq protein ID)** |
| --- | --- | --- | --- | --- | --- | --- | --- |
|  |  |  | **Expression** | **Highest in** |  |  |  |
| *RFX1* | pA@RFX1 | chr19:14117085-14117141,- | Broad | cerebellum (brain) | ENST00000254325 (NM_002918) | - | ENSP00000254325  (NP_002909) |
|  | pB@RFX1 | chr19:14117148-14117157,- |  |  |  |  |  |
|  | pC@RFX1 | chr19:14117622-14117633,- | Specific | testis | **Novel**  **pC@RFX1 transcript** | Alternative in-frame ATG in Exon 3 of ENST00000254325 | A possible novel protein with a shorter N-terminal from ENSP00000254325 |
| *RFX2* | pA@RFX2 | chr19:6110474-6110551,- | Broad | uterus | ENST00000303657 (NM_000635) | - | ENSP00000306335 (NP_000626) |
|  | pB@RFX2 | chr19:6110555-6110617,- |  | testis |  |  |  |
|  | pC@RFX2 | chr19:6110458-6110472,- |  | medulla oblongata (brain) |  |  |  |
| *RFX3* | pA@RFX3 | chr9:3525867-3526016,- | Broad | cerebellum (brain) | ENST00000382004 (NM_134428) | - | ENSP00000371434 (NP_602304) |
|  | pB@RFX3 | chr9:3525727-3525828,- |  | lung, fetal |  |  |  |
|  | pC@RFX3 | chr9:3526360-3526421,- |  | cerebellum (brain) | **Novel**  **pC@RFX3 transcript** | Same ATG as ENST00000382004 |  |
|  | pD@RFX3 | chr9:3525848-3525863,- |  | lung, fetal | ENST00000382004 (NM_134428) | - |  |
|  | pE@RFX3 | chr9:3489406-3489437,- | Specific | testis | **Novel**  **pE@RFX3 transcript** | Same ATG as ENST00000382004 |  |
| *RFX4* | pA@RFX4 | chr12:106976656-106976850,+ | Specific | spinal cord | ENST00000392842 (NM_213594) | - | ENSP00000376585 (NP_998759) |
|  | pB@RFX4 | chr12:107078493-107078533,+ | Specific | testis | ENST00000229387 (NM_032491) |  | ENSP00000229387 (NP_115880) |
|  | pC@RFX4 | chr12:106994905-106994954,+ |  |  | ENST00000357881 (NM_001206691) |  | ENSP00000350552 (NP_001193620) |
| *RFX5* | pA@RFX5 | chr1:151319710-151319774,- | Broad | blood (immune system) | ENST00000290524 (NM_000449, NM_001025603) | - | ENSP00000290524 (NP_000440, NP_001020774) |
|  | pB@RFX5 | chr1:151319654-151319698,- |  | tonsil (immune system) |  |  |  |
|  | pC@RFX5 | chr1:151319318-151319338,- |  | brain, fetal | **Novel**  **pC@RFX5 transcript** | Same ATG as ENST00000290524 |  |
|  | pD@RFX5 | chr1:151319283-151319314,- |  | duodenum, fetal (GI) | **Novel *(as pC@RFX5)*** | - |  |
| *RFX6* | pA@RFX6 | chr6:117198400-117198441,+ | Specific | duodenum, fetal (GI) | ENST00000332958 (NM_173560) | - | ENSP00000332208 (NP_775831) |
|  | pB@RFX6 | chr6:117198376-117198383,+ |  |  |  |  |  |
|  | pC@RFX6 | chr6:117198458-117198464,+ |  |  |  |  |  |
| *RFX7* | pA@RFX7 | chr15:56535946-56535987,- | Broad | cerebellum (brain) | **Novel**  **pA@RFX7 transcript** | Same ATG as ENST00000559447 | ENSP00000453281 (NP_073752) |
|  | pB@RFX7 | chr15:56535468-56535521,- |  |  | ENST00000559447 (NM_022841) | - |  |
|  | pC@RFX7 | chr15:56535722-56535764,- |  |  | **Novel**  **pC@RFX7 transcript** | Same ATG as ENST00000559447 |  |
|  | pD@RFX7 | chr15:56535922-56535936,- |  |  | **Novel *(as pA@RFX7)*** | - |  |
| *RFX8* | pA@RFX8 | chr2:102091566-102091581,- | Lowly expressed  (TPM < 5) | thymus (immune system) | **Novel**  **pA@RFX8 with DBD transcript**  **Novel**  **pA@RFX8 without DBD transcript** | pA@RFX8 validation reveals a long first exon, which includes the downstream pB@RFX8.  Evidence of alternative in-frame ATG in Exon 2 of ENST00000428343 and four variants of DBD splicing.  Based on the number of cDNA clones, we assume two novel transcripts: pA@RFX8 with and without DBD. | Two possible novel proteins, with and without DBD. |
|  | pB@RFX8 | chr2:102091144-102091183,- |  | medial frontal gyrus (brain) | ENST00000428343 (NM_001145664) |  | ENSP00000401536 (NP_001139136) without DBD. |
|  | pC@RFX8 | chr2:102091478-102091490,- | Noise  (TPM <1) | heart | **Novel, but not validated because TPM < 1** |  | - |
|  | pD@RFX8 | chr2:102091590-102091601,- |  | breast | **Novel *(as pA@RFX8)*** |  |  |
|  | pE@RFX8 | chr2:102091514-102091528,- |  | rectum, fetal | **Novel *(as pC@RFX8)*** |  |  |

Thirty TSS locations from eight human *RFX* genes and their respective tissue profile summaries are presented (cf. Materials and Methods). A given TSS location is considered as being expressed broadly if it is expressed at TPM > 5 in a large number and variety of tissues (n > 10). Conversely, a given TSS location is considered as being expressed specifically if it is expressed at TPM > 5 in a small number of tissues of the same organ (n < 10). The exceptions are: **(i)** pA@RFX4 displays high expression in many tissues (n > 10) but specifically in the brain and spinal cord; **(ii)** *RFX8* TSS locations are either lowly expressed (TPM < 5) or at background noise levels (TPM < 1). The highest tissue is the tissue with the highest TPM value from adult or otherwise indicated as fetal. GI means gastrointestinal tract. A given TSS is indicated as novel when it does not overlap with or is not found within +/-50 bp of the start site (indicated as exon 1) of the protein-coding transcripts with complete open reading frame description in the Ensembl database (release 81 – July 2015). Ensembl protein ID, as well as Refseq transcript and protein IDs were derived from the indicated Ensembl transcript ID (Ensembl BioMart tool). For the primers used in experimental validations and for verified transcript sequences, see Tables S8 and S9, respectively.

**Table S2: Positions of functional domains encoded by human *RFX* transcripts**

| **Gene Name** | **Transcript Name** | **TSS Reference** | **Transcript Length** | **5'UTR** | **5' UTR Length** | **Protein Domains** | | | | | | | | | | **3’ UTR** | **3' UTR Length** |
| --- | --- | --- | --- | --- | --- | --- | --- | --- | --- | --- | --- | --- | --- | --- | --- | --- | --- |
|  |  |  |  |  |  | **AD** | **AD Length** | **DBD** | **DBD Length** | **B** | **B Length** | **C** | **C Length** | **DIM** | **DIM Length** |  |  |
| *RFX1* | ENST00000254325 (RFX1-001) | pA | 4392 | 1-295 | 295 | 938-1423 | 486 | 1601-1831 | 222 | 2102-2215 | 114 | 2354-2464 | 111 | 2522-2974 | 453 | 3236-4392 | 1157 |
|  | pC@RFX1 transcript | pC | 3850 | 1-80 | 80 | 396-881 | 486 | 1068-1289 | 222 | 1560-1673 | 114 | 1812-1922 | 111 | 1980-2432 | 453 | 2694-3850 | 1157 |
| *RFX2* | ENST00000303657 (RFX2-002) | pA | 4065 | 1-222 | 222 | 340-663 | 324 | 820-1041 | 222 | 1312-1425 | 114 | 1570-1680 | 111 | 1738-2190 | 453 | 2395-4065 | 1671 |
| *RFX3* | ENST00000382004 (RFX3-006) | pA | 9359 | 1-364 | 364 | 476-757 | 282 | 914-1135 | 222 | 1406-1519 | 114 | 1667-1777 | 111 | 1835-2287 | 453 | 2615-9359 | 6745 |
|  | pC@RFX3 transcript | pC | 9678 | 1-683 | 683 | 795-1076 | 282 | 1233-1454 | 222 | 1725-1838 | 114 | 1986-2096 | 111 | 2154-2606 | 453 | 2934-9678 | 6745 |
|  | pE@RFX3 transcript | pE | 9192 | 1-197 | 197 | 309-590 | 282 | 747-968 | 222 | 1239-1352 | 114 | 1500-1610 | 111 | 1668-2120 | 453 | 2448-9192 | 6745 |
| *RFX4* | ENST00000392842 (RFX4-002) | pA | 3983 | 1-442 | 442 | - | - | 626-847 | 222 | 1028-1141 | 114 | 1217-1327 | 111 | 1385-1855 | 471 | 2651-3983 | 1333 |
|  | ENST00000229387 (RFX4-001) | pB | 3378 | 1-118 | 118 | - | - | - | - | 422-535 | 114 | 611-721 | 111 | 779-1249 | 471 | 2045-3378 | 1334 |
|  | ENST00000357881 (RFX4-003) | pC | 2574 | 1-149 | 149 | - | - | 360-581 | 222 | 762-875 | 114 | 951-1061 | 111 | 1119-1589 | 471 | 2385-2574 | 190 |
| *RFX5* | ENST00000290524 (RFX5-001) | pA | 3623 | 1-226 | 226 | - | - | 503-727 | 225 | - | - | - | - | - | - | 2078-3623 | 1546 |
|  | pC@RFX5 transcript | pC | 3643 | 1-246 | 246 | - | - | 523-747 | 225 | - | - | - | - | - | - | 2098-3643 | 1546 |
| *RFX6* | ENST00000332958 (RFX6-001) | pA | 3506 | 1-62 | 62 | - | - | 435-656 | 222 | 837-950 | 114 | 1026-1136 | 111 | 1194-1679 | 486 | 2850-3506 | 657 |
| *RFX7* | ENST00000559447 (RFX7-001) | pB | 10483 | 1-329 | 329 | - | - | 363-584 | 222 | - | - | - | - | - | - | 4422-10483 | 6062 |
|  | pA@RFX7 transcript | pA | 10470 | 1-316 | 316 | - | - | 350-571 | 222 | - | - | - | - | - | - | 4409-10470 | 6062 |
|  | pC@RFX7 transcript | pC | 10372 | 1-218 | 218 | - | - | 252-473 | 222 | - | - | - | - | - | - | 4311-10372 | 6062 |
| *RFX8* | ENST00000428343 (RFX8-001) | pB | 1704 | 1-138 | 138 | - | - | - | - | 307-405 | 99 | 481-591 | 111 | 649-1128 | 480 | 1561-1704 | 144 |
|  | pA@RFX8 with DBD transcript | pA | 2315 | 1-536 | 536 | - | - | 540-698 | 159 | 918-1016 | 99 | 1092-1202 | 111 | 1260-1739 | 480 | 2172-2315 | 144 |
|  | pA@RFX8 without DBD transcript | pA | 2166 | 1-540 | 540 | - | - | - | - | 769-867 | 99 | 943-1053 | 111 | 1111-1590 | 480 | 2023-2166 | 144 |

The 5’ UTR, protein domain and 3’ UTR positions and lengths are given in accordance with the respective transcript in nucleotides. The transcript names refer to the Ensembl transcript IDs unless otherwise indicated.

**Table S3: Experimentally proven, direct RFX target genes in humans from the literature**

| **FANTOM5 symbol** | **Direct RFX**  **target gene** | **Gene description** | **Reference** |
| --- | --- | --- | --- |
| *ALMS1* | *ALMS1* | Alstrom syndrome protein 1, centrosome and basal body associated protein | Purvis *et al*, 2010 |
| *CD70* | *CD70* | CD70 molecule | Zhao *et al*, 2010 |
| *COL1A2* | *COL1A2* | collagen type I alpha 2 chain | Sengupta *et al*, 2002 |
| *DCDC2* | *DCDC2* | doublecortin domain containing 2 | Tammimies *et al*, 2016 |
| *DYX1C1* | *DYX1C1* | dyslexia susceptibility 1 candidate 1 | Tammimies *et al*, 2016 |
| *FGF1* | *FGF1 (1B promoter)* | fibroblast growth factor 1 | Hsu *et al*, 2010 |
| *GPR56* | *GPR56* | G protein-coupled receptor 56 | Bae *et al*, 2014 |
| *HLA-B* | *HLA-B* | major histocompatibility complex, class I, B | Neerincx *et al*, 2012 |
| *HLA-DRA* | *HLA-DRA* | major histocompatibility complex, class II, DR alpha | Reith *et al*, 1988 |
| *IL5RA* | *IL-5Rα* | interleukin 5 receptor subunit alpha | Iwama *et al*, 1999 |
| *INS* | *INS* | insulin | Chandra *et al*, 2014 |
| *ITGAL* | *CD11a* | integrin subunit alpha L | Zhao *et al*, 2010 |
| *KIAA0319* | *KIAA0319* | neuronal migration, dyslexia-associated protein | Tammimies *et al*, 2016 |
| *KIF3A* | *KIF3A* | kinesin family member 3A | Kang *et al*, 2015 |
| *MAP1A* | *MAP1A* | microtubule associated protein 1A | Nakayama *et al*, 2003 |
| *MYC* | *c-myc* | v-myc avian myelocytomatosis viral oncogene homolog | Chen *et al*, 2000 |
| *PADI1* | *PADI1* | peptidyl arginine deiminase 1 | Adoue *et al*, 2008 |
| *PTPN6* | *SHP1* | protein tyrosine phosphatase, non-receptor type 6 | Amin *et al*, 2011 |
| *RFX1* | *RFX1* | regulatory factor X1 | Lubelsky *et al*, 2005 |
| *TGFB2* | *TGFβ2* | transforming growth factor beta 2 | Feng and Zuo, 2012 |
| *TMEM138* | *TMEM138* | transmembrane protein 138 | Lee *et al*, 2012 |
| *TMEM216* | *TMEM216* | transmembrane protein 216 | Lee *et al*, 2012 |

Direct target genes of human RFX TFs were considered as being confirmed when a biochemical interaction between RFX TF and the respective X-box promoter motif has been shown experimentally or when X-box function was demonstrated by mutation analysis.

| **JASPAR ID** | **JASPAR Name** | **JASPAR Evidence** | **Own Description** | **JASPAR Sequence Logo and Frequency Matrix** |
| --- | --- | --- | --- | --- |
| MA0600.1 | RFX2 | ChiP-seq | The full X-box motif consisting of two imperfect inverted repeats, or half sites, of 6 nt each that are joined by a 2 nt spacer (AT). | A [ 373 62 192 431 34 1 1567 164 581 193 214 2095 2312 0 760 588 406 585 620 ]  C [ 141 184 736 317 1936 2162 432 222 0 5 1932 8 0 2294 784 403 831 630 668 ]  G [1716 317 64 910 121 7 41 94 1765 2146 0 170 28 0 539 1178 733 761 678 ]  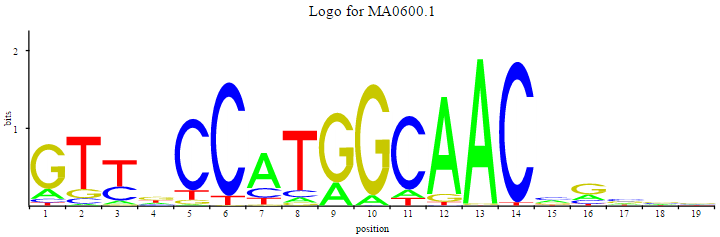T [ 116 1783 1354 688 255 176 306 1866 0 2 200 73 6 52 263 177 376 370 380 ] |
| MA0510.1 | RFX5 | ChiP-seq | The 3’ half site of the X-box. | A [ 343 595 6 181 1189 0 1844 245 0 3057 3692 0 2084 956 871 ]  C [1572 796 1870 3687 2171 832 1 0 2815 497 176 3868 782 361 1388 ]  G [ 561 862 1283 0 61 0 2023 3514 408 314 0 0 0 2184 1239 ]  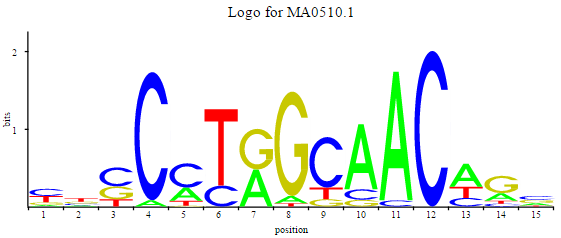T [1392 1615 709 0 447 3036 0 109 645 0 0 0 1002 367 370 ] |

**Table S4: Human X-box motifs selected from the JASPAR database**

Source: <http://jaspar.genereg.net/>

**Table S5: Experimentally validated human X-box motif sequences in promoter regions that were captured by the scanning criteria**

| **Direct RFX**  **target gene** | | **Validated X-box motif sequence** | **X-box position to the TSS** | **JASPAR profile** |
| --- | --- | --- | --- | --- |
| *MYC*  (c-myc) | | **GTAG**T**T** AT **GGTAAC** TGGGG | +522 | MA0600.1 (RFX2) |
| *FGF1* | | **GTT**T**CC** CT **GGCAAC** TCAGG | -447 | MA0600.1 (RFX2) |
| *COL1A2* | | AG**CACC** AC **GGCA**G**C** AGGAG | +8 | MA0600.1 (RFX2) |
| *ALMS1* | | **GTC**C**CT** **AGCAAC** GCG  GC **GTC**C**CT** **AGCAAC** GCGCG | -90  -92 | MA0510.1 (RFX5)  MA0600.1 (RFX2) |
| *CD70* | | CATC CT **GGCAAC** TGC  TG**CA**T**C** CT **GGCAAC** TGCCT | -100  -102 | MA0510.1 (RFX5)  MA0600.1 (RFX2) |
| *IL5RA*  (*IL-5Rα*) | | G T**TG**C**CT** A **GG**AG**AC** AGAGG | -424 | MA0600.1 (RFX2) |
| *MAP1A* | 5’ | **GTT**T**CC** AT **GG**AG**AC** CGAGG  **GTC**T**CC** AT **GG**A**AAC** ACCCG | +38 (1)  +33 (-1) | MA0600.1 (RFX2)  MA0600.1 (RFX2) |
|  | 3’ | **GTTGCC** AT **GG**AG**AC** AACTC  **GTC**T**CC** AT **GGCAAC** GCCGT | +80 (1)  +75 (-1) | MA0600.1 (RFX2)  MA0600.1 (RFX2) |
| *HLA-DRA* | | C**TT**C**CC** CT **AGCAAC** AGATG | -92 | MA0600.1 (RFX2) |
| **X-box sequence logo** | | 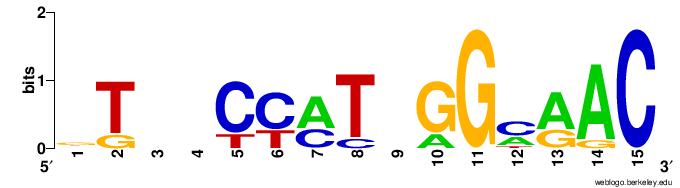 |  |  |

Sequences shown in bold fit to the X-box consensus motif GTNRC(C/N) – N_0–3_ – RGYAAC as described by Emery *et al*. (1996). The X-box position is on the coding strand, except for *MAP1A* with X-boxes on both coding (1) and non-coding (-1) strands. The X-box sequences shown in this Table were captured using the scanning criteria of JASPAR 80% threshold and independently with the MEME FIMO motif scan tool (Grant *et al*, 2011) with a p-value < 0.0001 using both JASPAR motifs (cf. Table S4) as queries. The sequence logo from the X-box motifs shown in this Table was made by WebLogo Version 2.8.2 (Crooks *et al*, 2004).

**Table S6: Experimentally validated human X-box motif sequences that were either in distal regions or that were not captured by the scanning criteria**

| **Direct RFX**  **target gene** | **Validated X-box motif sequence** | **X-box position as indicated in the literature** |
| --- | --- | --- |
| *PADI1* | TTGA **GTT**C**CT** **G**AGG**AC** CCA | Long range^1^ |
| *PTPN6*^2^  (*SHP1*) | CT **G**T**T**GC**C** CA  CT **G**C**C**GC**C** CA | -706 to -676 of promoter |
| *TGFB2* | **GTGG**T**C** TA **AGTAAC** | -113 to -100 of TSS |
| *ITGAL*  (*CD11a*) | CA**G**C**CT** **GTT**GC**C** TCTGTGAGA | -55 to -37 of TSS |
| *TMEM216* | G AA**G**C**CA** **GCTAAC** (RE1)  **GTTACC** **A**A**C**C**AC** (RE2) | Intergenic region^3^ |
| *TMEM138* |  |  |
| *HLA-B*^4^ | C CA **GGA**T**AC** TCG | MHC I promoter |
| *RFX1^5^*  (distal) | CAGTTG CC  (rev-compl) **GGCAAC** TG | Within -355 to +1 of the TSS |
| *RFX1*  (proximal) | **GGCAAC** GC |  |
| **X-box sequence logo** | 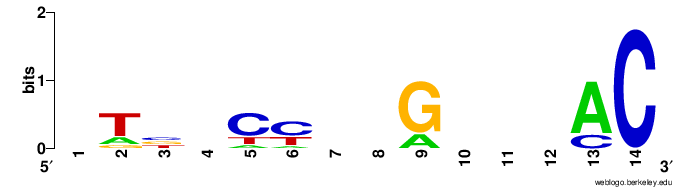 |  |

Sequences shown in bold fit to the X-box consensus motif GTNRC(C/N) – N_0–3_ – RGYAAC as described by Emery *et al.* (1996). The X-box sequences shown in this Table, if they are stated to be in the promoter region, were *not* captured using the scanning criteria of JASPAR 80% threshold and independently with the MEME FIMO motif scan tool (Grant *et al*, 2011) with a p-value < 0.0001 using both JASPAR motifs (cf. Table S4) as queries. The sequence logo from the X-box motifs shown in this Table was made by WebLogo Version 2.8.2 (Crooks *et al*, 2004).

1. The authors indicate the X-box motif as an MIBP1/RFX1 binding site, located approximately 38 kb upstream of the gene.
2. The authors used TT (grey shaded letters) instead of CC in the reference genome, which may be due to common SNPs. The X-box is approximately 5.5 kb upstream of the TSS.
3. Two regulatory elements (RE1 and RE2), which contain the X-box motifs, are found in the intergenic region between *TMEM216* (downstream) and *TMEM138* (upstream). The identified X-box motifs locate at a 10-15 kb distance from these genes.
4. The authors indicate the X-box motif as the X1 element in the *HLA* class I B gene promoter.
5. The reverse complement sequence was the one that fit the X-box consensus motif.

**Table S7: *RFX* correlated enhancers within +/- 500 kb of *RFX* TSS locations**

| ***RFX* gene** | ***RFX* TSS** | ***RFX* correlated enhancer** | **correlation** | **p-value** | **FDR** |
| --- | --- | --- | --- | --- | --- |
| *RFX2* | pA@RFX2 | chr19:6063576-6063994 | 0.147954067 | 9.44E-06 | 0.000886461 |
|  | pA@RFX2 | chr19:6071128-6071397 | 0.154597629 | 3.64E-06 | 0.000379891 |
|  | pA@RFX2 | chr19:6108978-6109271 | 0.135315057 | 5.18E-05 | 0.003738885 |
|  | pA@RFX2 | chr19:6111447-6111675 | 0.142178616 | 2.09E-05 | 0.00163668 |
| *RFX4* | pB@RFX4 | chr12:106664099-106664223 | 0.1821097 | 4.55E-08 | 6.11E-06 |
|  | pB@RFX4 | chr12:107363542-107363991 | 0.192071672 | 7.81E-09 | 3.66E-06 |
|  | pB@RFX4 | chr12:107396424-107396642 | 0.185696996 | 2.44E-08 | 5.73E-06 |
|  | pB@RFX4 | chr12:107494078-107494256 | 0.182332225 | 4.38E-08 | 6.11E-06 |
| *RFX7* | pC@RFX7 | chr15:57016270-57016644 | 0.194337655 | 5.16E-09 | 3.66E-06 |
|  | pD@RFX7 | chr15:56063154-56063228 | 0.182701878 | 4.11E-08 | 6.11E-06 |
|  | pD@RFX7 | chr15:56127522-56127639 | 0.188505108 | 1.48E-08 | 4.64E-06 |
|  | pD@RFX7 | chr15:56162319-56162688 | 0.164444751 | 8.23E-07 | 9.66E-05 |
| *RFX8* | pD@RFX8 | chr2:102399741-102400097 | 0.146885326 | 1.10E-05 | 0.000935819 |

These 13 candidate *RFX* enhancers were located within -500 kb to +500 kb of *RFX* TSS locations as extracted from Andersson *et al* (2014) and whose expressions were significantly correlated (Spearman correlation with multiple testing correction, False Discovery Rate < 0.05) with the *RFX* TSS locations based on FANTOM5 CAGE expression values (TPM) in 889 biological samples.

**Table S8: Primer sequences for novel *RFX* transcripts validation**

| ***RFX* transcript** | **Primers (5’🡪3’)** |
| --- | --- |
| pC@RFX1 | F, CACCGAGCGAGAGAGAAGTCTG  R, CTGAGTAGGAACGCCGGTCT |
| pC@RFX3 | F, GCCATAGCTCGTTTCTTCAC  R, CAGTATCGCTTCCTTCCACA |
| pE@RFX3 | F, TGTGAACTTCTGCTTCCACTAC  R, CTTCCACATACTGCACCTGAG |
| pC@RFX5 | F, GTCGTGGCGAGATTAAGTAATG  R, CCTCTACTTTGTTCTGCACGG |
| pA@RFX7 | F, TGGGGAGTGGCGTGGAG  R, CATGCATTTGTTGTGCCCGA |
| pC@RFX7 | F, CGCCATGCCTCTCCCG  R, CTCCTGGCAAAGGGGATTGT |
| pA@RFX8 | F, aggaagtgaacttggagatgg  R, CCACAGGTCTCCACGTAAATC |
| RFX8 DBD | F, GCATTTGCGAAGAGTGCAGC  R, GCAGTATTCGTCAGCAAGGAAG |

**Table S9: Verified novel *RFX* transcript sequences**

| ***RFX* transcript** | **Verified transcript sequence** | **cDNA source (TPM)** | **cDNA evidence (#clones)** |
| --- | --- | --- | --- |
| pC@RFX1 | **CACCGAG**CGAGAGAGAAGTCTGGAGCGGAGTCTTCCTTTCTCGCCTTTTTAATTTGAAGGTGCCATGCGGGCCAGCGAGACAGTGTCGGAGGCCAGCCCCGGCTCCACCGCCAGCCAGACCGGCGTTCCTACTCAG | Testis  (19.36) | 1 |
| pC@RFX3 | **GCCATAGCTCGTTTCTTCACAAAGTGC**CTGGAATCACCCTCGCCACCCCCTCCCCCGGGTTAGCCGTTACTCTCTCGTGGCTCCGGGACGTCTCTGTACAAAGCTGGCTGCTGGGACAGGCAGCCCTCGTTTCACAGATAGTACCCCTCGCACCACGACTCCTGGGCCTCGCCAGCGCGTGTAGTAACGTCCCTCCGAGAGGACTCTTGGTGACCTCAGCCCCAGCCAAGCATAGGCGCACCTCTTCGGAGTCCTGCGGTGGAATCCCGGCCTCGTGCCCCGTCACCCCGCCGCCCCGCCACCCCACCCGGGTTTGGGGGCCGTCAGTCACAGCGCTGCGCCCTCCCCTTCCGGCGGCTTGGCCGGGCCCGCCTCCTCCCCTCTTCCCTCCCCCACCCCTCCCCGTGCGAGTGTCTCCCTCTCCCTCTCGCTCTCTCTCTCTCTCCCTCTCTCTCTCTCTTTTGTGAGTTATAGCAACCGTTGCCTTGTGAATCgAGCGCCATAGTCACCGTAGTCCTGGCGACTGTcACCCATCAACAACAACTACTCCTCaCCTCCTCCTCCTCCTCTTCCTCCTtCCTCCTCCCCACtACCACCATCTCCATCACCCACCAACAACACCACAATAATCCACAGCCAAGAGACCATCATGCAGACATCAGAGACTGGGTCGGACACAGGCTCGACAGTGACCTTACAAACATCTGTGGCTAGTCAAGCAGCAGTGCCTACGCAGGTGGTACAGCAAGTACCAGTACAACAACAGGTgCAGCAGGTACAGACTGTGCAGCAGGTACAACATGTCTATCCCGCTCAGGTGCAGTATGTGGAAGGAAGCGATACTG | Whole brain^1^  (89.37) | 4 |
| pE@RFX3 | **TGTGAACTTCTGCTTCCACTACAAACAG**TACTTTAAAGACAGGCCTTCCACTGCCATTCAGAAAGCAGCCATGGAGGATACTCTAGTTTCTTTGGGAAGATAAACACTCAATCTGCTAGTTAAGAGACAAAGCTCAAGAGAAATAAGCTGAAGCTGTAACCTCCATTTCAAACTTGTGGTTCTGCAGACCATCATGCAGACATCAGAGACTGGGTCGGACACAGGCTCGACAGTGACCTTACAAACATCTGTGGCTAGTCAAGCAGCAGTGCCTACGCAGGTGGTACAGCAAGTACCAGTACAACAACAGGTACAGCAGGTACAGACTGTGCAGCAGGTACAACATGTCTATCCCGCTCAGGTGCAGTATGTGGAAG | Testis  (19.91) | 2 |
| pC@RFX5 | **GTCGTGGCGAGATTAAGT**AATGAGAACTTGGGCCCAGTcTTTTTTCCAAGCCTAGAAGGGCAGAATATGTTCGTATCCTTTCTGACCTCCCAGGCATATGCAATGTTTTCCAGATTTAGGAGACTTCAGAAAGGTGGGGCAGATAGAATGGAGATGGCAAAGATCTCTTTGGGCATATATGGGCCTGGCGAAGTAATGGAATAATTTCTAATTTTCGGAGAAGCCCTCATGCCGGGATGGCAGAAGATGAGCCTGATGCTAAGAGCCCCAAGACTGGGGGAAGGGCCCCCCCAGGTGGTGCTGAGGCTGGGGAACCTACCACCCTTCTTCAGAGGCTCCGAGGTACCATTTCCAAGGCCGTGCAGAACAAAGTAGAGG | Whole brain^1^  (27.08) | 1 |
| pA@RFX7 | **TGGGGAGTGGCGTGGAGCG**ATGGCAGAGGAACAACAACAGCCGCCACCACAGCAGCCTGATGCCCATCAGCAGCTTCCCCCCAGCGCCCCCAACTCGGGGGTGGCCCTGCCAGCCCTTGTGCCCGGGCTGCCAGGGACAGAGGCCAGCGCGCTGCAACACAAGATCAAGAACTCCATCTGCAAAACTGTACAATCTAAAGTGGACTGCATTTTGCAAGAAGTTGAGAAGTTTACAGACCTAGAGAAACTCTACCTCTACCTTCAGCTGCCTTCTGGTCTCAGCAATGGAGAGAAAAGTGATCAGAATGCCATGTCATCTAGTCGGGCACAACAAATGCATG | Whole brain^1^  (290.52) | 2 |
| pC@RFX7 | **CGCCATGCCTCTCCCGCGGTGAAGCG**CCCCGGCCGTGAGGAGCCGCTGGTCTCCCCGGTGATGTTCCCCAGGCGGCAGGCGAAAGCGACTCACTCGAGCCCTGGGCGATGGCAGAGGAACAACAgCAGCtCACCACctaatggtgactcaatcaataaagaccctaaattatgcactaaaagcccaagaaaacgactgtcttctacattgcaagagacccaggtgcctcctgtaaagaaaccaattgtggaacagctttcagcagctaccatagaagggcagaaacaaggcagtgttaagaaggaccaaaaggttccacattcagggaaaacagaaggttcaacagcaggtgctcagattcctagcaaggtatcagtaaATGTCATCTAGTCGGGCACAACAAATGCATGCCTTTTCCTGGATTCGGAATACCCTAGAGGAACATCCGGAGACTTCACTGCCCAAACAGGAAGTCTATGAcGAGTACAAGAGCTATTGTGACAATCTTGGTTACCATCCATTAAGTGCTGCTGATTTTGGAAAGATCATGAAAAACGTCTTTCCAAACATGAAGGCACGTCGTTTGGGCACAAGAGGgCAAATCTAAATATTGCTACAGTGGAaTAAGAAAAAAAGCTTTTGTTCATATGCCAACACTGCCCAACCTTGACTTTCACAAAACTGGAGATGGGTTGGAAGGAGCTGAACCTTCTGGGCAGCTTCAAAATATTGATGAAGAAGTTATCTCTTCTGCTTGCCGTCTTGTGTGTGAGTGGGCCCAGAAAGTGTTAAGCCAACCATTTGACACCGTCTTGGAATTAGCCCGCTTCCTTGTAAAAAGTCACTATATAGGCACCAAGTCAATGGCAGCTCTAACTGTAATGGCAGCAGCACCAGCAGGAATGAAAGGAATTACCCAGCCTTCTGCTTTTATACCTACAGCTGAAAGTAATTCCTTTCAGCCTCAGGTGAAGACTTTGCCATCTCCAATTGATGCTAAACAGCAGTTGCAACGGAAAATCCAGAAGAAGCAGCAAGAtCAGAAACTACAATCCCCTTTGCCAGGAG | Whole brain^1^  (159.9) | 2  Repeat regions between the TSS and the ATG were difficult to sequence. |
| pA@RFX8 | **AGGAA**GTGAACTTGGAGATGGCCAGCAGCTCCATGTCACACAGCAATGACACTGACATAAATGGCTCTTGAGAGCAAGGACTGGCAGGACCTGGCTACAGATATGGGGACTCACGTTGAGGTGGCACTGCTCACCTGGAGCAGCACACTGGAGTCACCTGGGACCAGCCTCCTAGAAGTCCAGATGCGCCCGCCTGCCAGAGAGCTCCGAGATTTTTCAAAACTCCTAGAGACAGTGGTTTTCAGCAACGGCGGGCACATTAGCATCACCCGGGACCCTGAAAAAGCCGGTTGGCCCAGGCGGCCCTCGCCCAGACCAATTCATCAGAACCTCTTGGGGTGGGGTGGGGCGGGGCTGGGATGGGGAGCGGCAGCCGGGGTATCAATA***TTTTTGAAAACTGCCTGAATAACTCCAACGCTCAAGCAA***GTCAAGGACACCCACGGACTCAACACCGCGACCAGATTGGAAAAGGTGTTGGTCGACAACTTCTGCATTTGCGAAGAGTGCAGCGTCCCTCGCTGTCTCATGTATGAGATTTACGTGGAGACCTGTGG (in ***bold italic*** is pB@RFX8) | Whole brain^1^  (4.33) | 2 |
| RFX8 DBD (chr2:102067115-102083307,-) | Out of 107 white colonies selected by PCR, 16 were chosen for sequencing based on colony PCR band sizes, which were larger than the ones of an empty vector.  Four DBD transcript variants were found.  Variants 1-3 would lead to downstream B, C, DIM domains translation.  Variant 1 corresponds to ENST00000428343 transcript that lacks the DBD.  Variant 2 would lead to downstream B, C, DIM domains translation when the alternative in-frame +1 ATG is used.  Variant 4 would lead to a premature STOP codon for all the possible frames after the DBD translation.  >RFX8 DBD variant 1  GCATTTGCGAAGAGTGCAGCGTCCCTCGCTGTCTCATGTATGAGATTTACGTGGAGACCTGTGGGCAAAACACTGAGAACCAAGTCAACCCGGCCACCTTTGGGAAGTGTGAAGATCATTCACCGATGAAGACAGACCCAGTTGGATCCCCTTTGTCTGAATTCAGGAGATGTCCATTTCTGGAGCAAGAACtGGCAAAGAAATACTCCTGTAATATGATGGCCTTCCTTGCTGACGAATACTGC  >RFX8 DBD variant 2  GCATTTGCGAAGAGTGCAGCGTCCCTCGCTGTCTCATGTATGAGATTTACGTGGAGACCTGTGGGCAAAACACTGAGAACCAAGTCAACCCGGCCACCTTTGGGAAGTGGAGATGCCATTGCCTTTGAAAAATCTACTAATTATAACAGCATTATCCAACAAGAAGCAACATGTGAAGATCATTCACCGATGAAGACAGACCCAGTTGGATCCCCTTTGTCTGAATTCAGGAGATGTCCATTTCTGGAGCAAGAACAGGCAAAGAAATACTCCTGTAATATGATGGCCTTCCTTGCTGACGAATACTGC  >RFX8 DBD variant 3  GCATTTGCGAAGAGTGCAGCGTCCCTCGCTGTCTCATGTATGAGATTTACGTGGAGACCTGTGGGCAAAACACTGAGAACCAAGTCAACCCGGCCACCTTTGGGAAGCTTGTGAGATTGGTTTTTCCaGACCTTGGCACCCGGtGGCTGGGCACTAGAGGAAGTGCCAGGTATCATTATGATGGAATCTGTATCAAGAAAAGCTCTTTCTTCTATGCCCAGTATTGCTgCCTGATAGGTGAAAAAAGGTATCACAGTGGAGATGCCATTGCCTTTGAAAAATCTACTAATTATAACAGCATTATCCAACAAGAAGCAACATGTGAAGATCATTCACCGATGAAGACAGACCCAGTTGGATCCCCTTTGTCTGAATTCAGGAGATGTCCATTTCTGGAGCAAGAACtGGCAAAGAAATACTCCTGTAATATGATGGCCTTCCTTGCTGACGAATACTGC  >RFX8 DBD variant 4  GCATTTGCGAAGAGTGCAGCGTCCCTCGCTGTCTCATGTATGAGATTTACGTGGAGACCTGTGGGCAAAACACTGAGAACCAAGTCAACCCGGCCACCTTTGGGAAGCTTGTGAGATTGGTTTTTCCGGACCTTGGCACCCGGAGGCTGGGCACTAGAGGAAGTGCCAGGTATCATTATGATGGAATCTGTATCAAGAAAAGCTCTTTCTTCTATGCCCAGTATTGCTgCCTGATAGGTGAAAAAGGTATCACAGATGGCCTTCCTTGCTGACGAATACTGC  Combining pA@RFX8 with two RFX8 DBD variants with the most number of cDNA clone evidence.  (The TSS is assumed to be pA@RFX8)  >pA@RFX8 without DBD transcript (pA@RFX8 to ATG + RFX8 DBD variant 2)  **AGGAA**GTGAACTTGGAGATGGCCAGCAGCTCCATGTCACACAGCAATGACACTGACATAAATGGCTCTTGAGAGCAAGGACTGGCAGGACCTGGCTACAGATATGGGGACTCACGTTGAGGTGGCACTGCTCACCTGGAGCAGCACACTGGAGTCACCTGGGACCAGCCTCCTAGAAGTCCAGATGCGCCCGCCTGCCAGAGAGCTCCGAGATTTTTCAAAACTCCTAGAGACAGTGGTTTTCAGCAACGGCGGGCACATTAGCATCACCCGGGACCCTGAAAAAGCCGGTTGGCCCAGGCGGCCCTCGCCCAGACCAATTCATCAGAACCTCTTGGGGTGGGGTGGGGCGGGGCTGGGATGGGGAGCGGCAGCCGGGGTATCAATATTTTTGAAAACTGCCTGAATAACTCCAACGCTCAAGCAAGTCAAGGACACCCACGGACTCAACACCGCGACCAGATTGGAAAAGGTGTTGGTCGACAACTTCTGCATTTGCGAAGAGTGCAGCGTCCCTCGCTGTCTCATGTATGAGATTTACGTGGAGACCTGTGGGCAAAACACTGAGAACCAAGTCAACCCGGCCACCTTTGGGAAGTGGAGATGCCATTGCCTTTGAAAAATCTACTAATTATAACAGCATTATCCAACAAGAAGCAACATGTGAAGATCATTCACCGATGAAGACAGACCCAGTTGGATCCCCTTTGTCTGAATTCAGGAGATGTCCATTTCTGGAGCAAGAACAGGCAAAGAAATACTCCTGTAATATGATGGCCTTCCTTGCTGACGAATACTGC  >pA@RFX8 with DBD transcript (pA@RFX8 to ATG + RFX8 DBD variant 3)  **AGGAA**GTGAACTTGGAGATGGCCAGCAGCTCCATGTCACACAGCAATGACACTGACATAAATGGCTCTTGAGAGCAAGGACTGGCAGGACCTGGCTACAGATATGGGGACTCACGTTGAGGTGGCACTGCTCACCTGGAGCAGCACACTGGAGTCACCTGGGACCAGCCTCCTAGAAGTCCAGATGCGCCCGCCTGCCAGAGAGCTCCGAGATTTTTCAAAACTCCTAGAGACAGTGGTTTTCAGCAACGGCGGGCACATTAGCATCACCCGGGACCCTGAAAAAGCCGGTTGGCCCAGGCGGCCCTCGCCCAGACCAATTCATCAGAACCTCTTGGGGTGGGGTGGGGCGGGGCTGGGATGGGGAGCGGCAGCCGGGGTATCAATATTTTTGAAAACTGCCTGAATAACTCCAACGCTCAAGCAAGTCAAGGACACCCACGGACTCAACACCGCGACCAGATTGGAAAAGGTGTTGGTCGACAACTTCTGCATTTGCGAAGAGTGCAGCGTCCCTCGCTGTCTCATGTATGAGATTTACGTGGAGACCTGTGGGCAAAACACTGAGAACCAAGTCAACCCGGCCACCTTTGGGAAGCTTGTGAGATTGGTTTTTCCaGACCTTGGCACCCGGtGGCTGGGCACTAGAGGAAGTGCCAGGTATCATTATGATGGAATCTGTATCAAGAAAAGCTCTTTCTTCTATGCCCAGTATTGCTgCCTGATAGGTGAAAAAAGGTATCACAGTGGAGATGCCATTGCCTTTGAAAAATCTACTAATTATAACAGCATTATCCAACAAGAAGCAACATGTGAAGATCATTCACCGATGAAGACAGACCCAGTTGGATCCCCTTTGTCTGAATTCAGGAGATGTCCATTTCTGGAGCAAGAACtGGCAAAGAAATACTCCTGTAATATGATGGCCTTCCTTGCTGACGAATACTGC | Whole brain^1,2^  (4.33,  *as pA@RFX8*) | 1 out of 16  3 out of 16  4 out of 16  1 out of 16 |

cDNA source: cDNA synthesis from Clontech Human Total RNAs: **(i)** Testis (Cat. No. 636533, Lot No. 1012048A, normal, pooled from 39 Caucasians, age: 14-64; sudden death); **(ii)** Whole brain^1^ (Cat. No. 636530, Lot No. 5080281, normal, from 43-yr-old Caucasian, male, sudden death) and **(iii)** Whole brain^2^ (Cat. No. 636530, Lot No. 1402002, normal, pooled from 4 males Asian, age: 21-29, cause of death N/A). TPM (tags per million) is the arbitrary unit for detection of expression as defined by FANTOM5 (FANTOM Consortium *et al*, 2014). We consider TPM < 5 to be lowly expressed and TPM < 1 to be background noise.

Verified transcript sequences: Underlined are the forward and reverse primers. Orange text denotes TSS sequences that were verified by the forward primer, respectively. Lower case lettering indicates discrepancies between what was verified by Sanger sequencing and cDNA sequences as identified by UCSC Genome Browser (GRCh37/hg19) BLAT tool. The reference start ATGs are shaded in green. The alternative start ATGs are shaded in yellow. For long cDNA products, overlapping verified sequences are shaded in grey. DBD exon regions for *RFX8* are shaded in red.

**Table S10: siRNA sequences for candidate RFX regulators**

Sequences of *SP2*, *ESR1* and scrambled (Scr) control siRNA molecules used in siRNA knockdown validation of candidate human RFX regulators. siRNAs were obtained from Eurofins Genomics: <https://www.eurofinsgenomics.eu/>.

| **siRNA** | **Sense sequence 5’🡪 3’**  **(dTdT 3’ not shown)** | **Optimal final concentration** | **%GC** | **Reference** |
| --- | --- | --- | --- | --- |
| *SP2* | 1)CGCUGUCUAAGACUAACAA  2)UGCAGACCAUCAACAUCAA | Mix 20nM  (10nM each) | 42.1  42.1 | 1) Zhao *et al*, 2014  2) Eurofins Genomics |
| *ESR1* | 1)UCAUCGCAUUCCUUGCAAA  2)GUAUUCAAGGACAUAACGA  3)GAUUGGCCAGUACCAAUGA | Mix 90nM  (30nM each) | 42.1  36.8  47.4 | 1) Hosey *et al*, 2007  2) Eurofins Genomics  3) Eurofins Genomics |
| Scr | AGGUAGUGUAAUCGCCUUG | 20nM for *SP2*  90nM for *ESR1* | 47.4 | Eurofins Genomics |

| **Type** | **Gene** | **Primer sequences (5’🡪3’)** | **Reference for sequence** | **Amplification efficiency (E)** |
| --- | --- | --- | --- | --- |
| siRNA site | *SP2* | F, CCCTCCCAGAACTTTCAGATC  R, GCTGCTACAGGTGGTGTTAG | Own design | 2.117576 |
| siRNA site | *ESR1* | F, ATCCTACCAGACCCTTCAGTG  R, GCCAGACGAGACCAATCATC | Ansems *et al*, 2015 | 2.34697 |
| Target | *RFX1* | F, AGAAGCAGTACGTGACGGAG  R, GCTGAACCACCTGAGTAGGAA | Own design | 2.657347 |
| Target | *RFX2* | F, CGCCGTCTACACCAATGGAG  R, CTCGAAGTAAGAAGCCGTGCTG | Own design | 1.964125 |
| Target | *RFX3* | F, TGGCGATTGAGACGCTGCAAA  R, TGGGAAGGCTCACTCCTTCTGCT | Own design | 2.009233 |
| Target | *RFX5* | F, GGCCGTGCAGAACAAAGTAG  R, CTCCTCATTGCTCAGTGTACTTG | Own design | 2.04346 |
| Target | *RFX7* | F, TGTGGCAGCTGTCCCTAGTCCC  R, TGAGGGGAAGAACTTTGCCGTCAG | Own design | 2.033581 |
| Reference gene | *HPRT1* | F, TCAGGCAGTATAATCCAAAGATGGT  R, AGTCTGGCTTATATCCAACACTTCG | Timper *et al*, 2011 | 2.031804 |
| Reference gene | *HSPCB* | F, AAGAGAGCAAGGCAAAGTTTGAG  R, TGGTCACAATGCAGCAAGGT | Fu *et al*, 2010 | 2.080983 |

**Table S11: qPCR primer sequences and amplification efficiencies for validation of candidate RFX regulators**

The qPCR primers used to measure mRNA expression levels after siRNA knockdown validation of candidate human *RFX* regulators. Primer pairs were designed to capture all the known transcript isoforms. Amplification efficiencies were determined by standard curve quantification with 4 serial dilutions (1ug, 500ng, 250ng, 125ng) in technical duplicates, using as template cDNA from MCF7 cells not treated with any type of siRNA.

**Figure S1: Human RFX1-8 DBD protein sequence alignment**


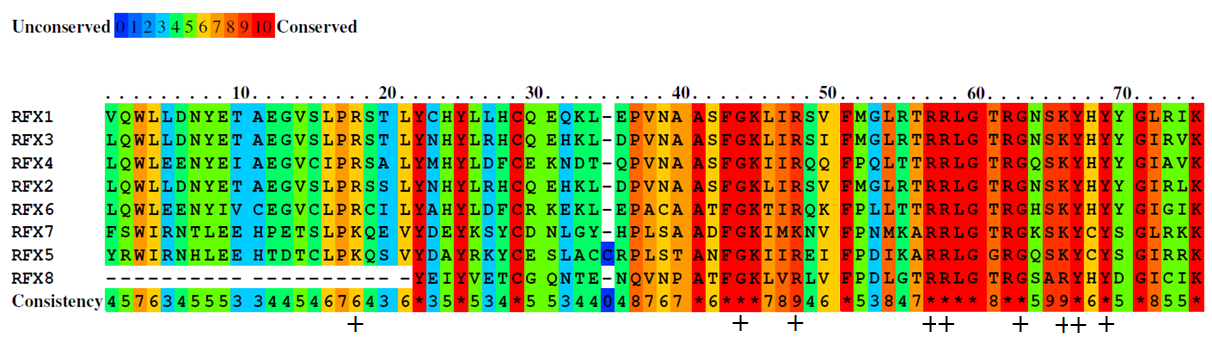


The multiple protein sequence alignment of the DNA binding domain (DBD) of human RFX1-8 by PRALINE tool with BLOSUM62 matrix (Simossis and Heringa, 2005: <http://www.ibi.vu.nl/programs/pralinewww/>). The nine DBD amino acid residues making direct or water-mediated DNA contacts (indicated by +) were determined by Gajiwala *et al* (2000).

**Supplementary references**

Adoue, V., Chavanas, S., Coudane, F., Méchin, M.-C., Caubet, C., Ying, S., Simon, M. (2008). Long-Range Enhancer Differentially Regulated by c-Jun and JunD Controls Peptidylarginine Deiminase-3 Gene in Keratinocytes. *Journal of Molecular Biology, 384*(5), 1048-1057.

Amin, S., Kumar, A., Nilchi, L., Wright, K., & Kozlowski, M. (2011). Breast Cancer Cells Proliferation Is Regulated by Tyrosine Phosphatase SHP1 through c-jun N-Terminal Kinase and Cooperative Induction of RFX-1 and AP-4 Transcription Factors. *Molecular Cancer Research, 9*(8), 1112-1125.

Andersson, R., Gebhard, C., Miguel-Escalada, I., Hoof, I., Bornholdt, J., Boyd, M., Sandelin, A. (2014). An atlas of active enhancers across human cell types and tissues. *Nature, 507*(7493), 455-461.

Ansems, M., Søndergaard, J., Sieuwerts, A., Looman, M. G., Smid, M., de Graaf, A. A., Adema, G. (2015). DC-SCRIPT is a novel regulator of the tumor suppressor gene CDKN2B and induces cell cycle arrest in ERα-positive breast cancer cells. *Breast Cancer Research and Treatment, 149*(3), 693-703.

Bae, B.-I., Tietjen, I., Atabay, K. D., Evrony, G. D., Johnson, M. B., Asare, E., Walsh, C. A. (2014). Evolutionarily Dynamic Alternative Splicing of GPR56 Regulates Regional Cerebral Cortical Patterning. *Science, 343*(6172), 764-768.

Bailey, T. L. & Elkan C. (1994). Fitting a mixture model by expectation maximization to discover motifs in biopolymers. *Proceedings of the Second International Conference on Intelligent Systems for Molecular Biology*, pp. 28-36, AAAI Press, Menlo Park, California, USA.

Chandra, V., Albagli-Curiel, O., Hastoy, B., Piccand, J., Randriamampita, C., Vaillant, E., Scharfmann, R. (2014). RFX6 Regulates Insulin Secretion by Modulating Ca2+ Homeostasis in Human β Cells. *Cell Reports, 9*(6), 2206-2218.

Chen, L., Smith, L., Johnson, M. R., Wang, K., Diasio, R. B., & Smith, J. B. (2000). Activation of Protein Kinase C Induces Nuclear Translocation of RFX1 and Down-regulates c-myc via an Intron 1 X Box in Undifferentiated Leukemia HL-60 Cells. *Journal of Biological Chemistry, 275*(41), 32227-32233.

Crooks, G. E., Hon, G., Chandonia, J.-M., & Brenner, S. E. (2004). WebLogo: A Sequence Logo Generator. *Genome Research, 14*(6), 1188-1190.

Emery, P., Strubin, M., Hofmann, K., Bucher, P., Mach, B., & Reith, W. (1996). A consensus motif in the RFX DNA binding domain and binding domain mutants with altered specificity. *Molecular and Cellular Biology, 16*(8), 4486-4494.

FANTOM Consortium, RIKEN PMI, & CLST (DGT). (2014). A promoter-level mammalian expression atlas. *Nature, 507*(7493), 462-470.

Feng, C., & Zuo, Z. (2012). Regulatory Factor X1-induced Down-regulation of Transforming Growth Factor β2 Transcription in Human Neuroblastoma Cells. *Journal of Biological Chemistry, 287*(27), 22730-22739.

Fu, J., Bian, L., Zhao, L., Dong, Z., Gao, X., Luan, H., Song, H. (2010). Identification of genes for normalization of quantitative real-time PCR data in ovarian tissues. *Acta Biochimica et Biophysica Sinica, 42*(8), 568-574.

Gajiwala, K. S., Chen, H., Cornille, F., Roques, B. P., Reith, W., Mach, B., & Burley, S. K. (2000). Structure of the winged-helix protein hRFX1 reveals a new mode of DNA binding. *Nature, 403*, 916-921.

Grant, C. E., Bailey, T. L., & Noble, W. S. (2011). FIMO: scanning for occurrences of a given motif. *Bioinformatics, 27*(7), 1017-1018.

Hosey, A. M., Gorski, J. J., Murray, M. M., Quinn, J. E., Chung, W. Y., Stewart, G. E., Harkin, D. P. (2007). Molecular Basis for Estrogen Receptor α Deficiency in BRCA1-Linked Breast Cancer. *Journal of the National Cancer Institute, 99*(22), 1683-1694.

Hsu, Y.-C., Liao, W.-C., Kao, C.-Y., & Chiu, I.-M. (2010). Regulation of FGF1 Gene Promoter through Transcription Factor RFX1. *Journal of Biological Chemistry, 285*(18), 13885-13895.

Iwama, A., Pan, J., Zhang, P., Reith, W., Mach, B., Tenen, D. G., & Sun, Z. (1999). Dimeric RFX Proteins Contribute to the Activity and Lineage Specificity of the Interleukin-5 Receptor α Promoter through Activation and Repression Domains. *Molecular and Cellular Biology, 19*(6), 3940-3950.

Kang, G. M., Han, Y. M., Ko, H. W., Kim, J., Oh, B. C., Kwon, I., & Kim, M.-S. (2015). Leptin Elongates Hypothalamic Neuronal Cilia via Transcriptional Regulation and Actin Destabilization. *Journal of Biological Chemistry, 290*(29), 18146-18155.

Lee, J. H., Silhavy, J. L., Lee, J. E., Al-Gazali, L., Thomas, S., Davis, E. E., Gleeson, J. G. (2012). Evolutionarily Assembled cis-Regulatory Module at a Human Ciliopathy Locus. *Science, 335*(6071), 966-969.

Moeenrezakhanlou, A., Shephard, L., Lam, L., & Reiner, N. E. (2008). Myeloid cell differentiation in response to calcitriol for expression CD11b and CD14 is regulated by myeloid zinc finger-1 protein downstream of phosphatidylinositol 3-kinase. *Journal of Leukocyte Biology, 84*(2), 519-528.

Nakayama, A., Murakami, H., Maeyama, N., Yamashiro, N., Sakakibara, A., Mori, N., & Takahashi, M. (2003). Role for RFX Transcription Factors in Non-neuronal Cell-specific Inactivation of the Microtubule-associated Protein MAP1A Promoter. *Journal of Biological Chemistry, 278*(1), 233-240.

Neerincx, A., Rodriguez, G. M., Steimle, V., & Kufer, T. A. (2012). NLRC5 Controls Basal MHC Class I Gene Expression in an MHC Enhanceosome-Dependent Manner. *The Journal of Immunology, 188*(10), 4940-4950.

Purvis, T. L., Hearn, T., Spalluto, C., Knorz, V. J., Hanley, K. P., Sanchez-Elsner, T., Wilson, D. I. (2010). Transcriptional regulation of the Alström syndrome gene ALMS1 by members of the RFX family and Sp1. *Gene, 460*(1–2), 20-29.

Reith, W., Satola, S., Sanchez, C. H., Amaldi, I., Lisowska-Grospierre, B., Griscelli, C., Mach, B. (1988). Congenital immunodeficiency with a regulatory defect in MHC class II gene expression lacks a specific HLA-DR promoter binding protein, RF-X. *Cell, 53*(6), 897-906.

Sengupta, P. K., Fargo, J., & Smith, B. D. (2002). The RFX Family Interacts at the Collagen (COL1A2) Start Site and Represses Transcription. *Journal of Biological Chemistry, 277*(28), 24926-24937.

Simossis, V. A., & Heringa, J. (2005). PRALINE: a multiple sequence alignment toolbox that integrates homology-extended and secondary structure information. *Nucleic Acids Research, 33*(suppl_2), W289-W294.

Suzuki, R., & Shimodaira, H. (2006). Pvclust: an R package for assessing the uncertainty in hierarchical clustering. *Bioinformatics, 22*(12), 1540-1542.

Tammimies, K., Bieder, A., Lauter, G., Sugiaman-Trapman, D., Torchet, R., Hokkanen, M.-E., Swoboda, P. (2016). Ciliary dyslexia candidate genes DYX1C1 and DCDC2 are regulated by Regulatory Factor (RF) X transcription factors through X-box promoter motifs. *The FASEB Journal, 30*(10), 3578-3587.

Timper, K., Grisouard, J., Radimerski, T., Dembinski, K., Peterli, R., Häring, A., Christ-Crain, M. (2011). Glucose-Dependent Insulinotropic Polypeptide (GIP) Induces Calcitonin Gene-Related Peptide (CGRP)-I and Procalcitonin (Pro-CT) Production in Human Adipocytes. *The Journal of Clinical Endocrinology & Metabolism, 96*(2), E297-E303.

Zhao, L., Yao, Y., Han, J., Yang, J., Wang, X., Tong, D., Shao, Y. (2014). miR-638 Suppresses Cell Proliferation in Gastric Cancer by Targeting Sp2. *Digestive Diseases and Sciences, 59*(8), 1743-1753.

Zhao, M., Sun, Y., Gao, F., Wu, X., Tang, J., Yin, H., Lu, Q. (2010). Epigenetics and SLE: RFX1 downregulation causes CD11a and CD70 overexpression by altering epigenetic modifications in lupus CD4+ T cells. *Journal of Autoimmunity, 35*(1), 58-69.

**---------------**
